# Physeal effects of posterior VBT are not uniform throughout a multi-tether construct in the kyphotic swine model

**DOI:** 10.1007/s43390-025-01247-0

**Published:** 2025-12-19

**Authors:** Matthew A. Halanski, Brittney Kokinos, Cameron Jeffers, Thomas Crenshaw

**Affiliations:** 1 Division of Orthopedics and Sports Medicine, Department of Child Health, Phoenix Children’s Hospital, University of Arizona College of Medicine, Phoenix, AZ USA; 2 Department of Animal Sciences, University of Wisconsin-Madison, Madison, WI USA

**Keywords:** Posterior vertebral body tethering (VBT), Growth modulation, Physis, Junctional kyphosis, Animal model

## Abstract

**Purpose:**

To assess if vertebral location within posterior VBT construct affected the overall vertebral growth rate, % growth modulation, and to determine if the proximal and distal physes within each tethered disk space responded similarly to tethering.

**Methods:**

Six hyper-kyphotic swine underwent multi-level posterior compressive tethering. Pulsed fluorochrome labeling was performed. Growth rates, % growth modulation, physeal zonal thicknesses, vertebral epiphyseal ossification, location of central nucleus pulposis, and vertebral shape by location within tether construct were measured.

**Results:**

Mean growth rates were similar throughout the vertebral levels studied and no significant difference was found between tethered or adjacent levels. However, the mean thickness of tethered physes was thinner than the adjacent uninstrumented physes (621 ± 36 μm vs 728 ± 52 μm, *p* = 0.001, adj *p* = 0.004) and this difference appeared to be primarily due to differences in the proliferative zone. The most proximal instrumented vertebral level, that corresponded anatomically to the 2nd and 3rd most distal thoracic level), had the greatest % growth modulation (55 ± 17%) compared to other instrumented levels (*p* = 0.0001, adj *p* value 0.004). No significant differences in growth rate, physeal thickness, or % growth modulation were found between the distal and proximal vertebral physes within each tethered disk. Adjacent level junctional kyphosis was observed through the significant reversal (negative) growth modulation at levels outside the construct (*p* < 0.0001, adj *p* value 0.004).

**Conclusion:**

Mean vertebral growth was not significantly inhibited by a posterior tether. Despite uniform tensioning throughout each construct, the most proximal tethered level experienced the most growth modulation indicating that growth may not be modulated the same at each level within a construct. Additionally, asymmetric vertebral growth may contribute to junctional kyphosis in the growing spine.

**Supplementary Information:**

The online version contains supplementary material available at 10.1007/s43390-025-01247-0.

## Introduction

Success of spinal deformity surgery is commonly measured by changes in the macroscale alignment of the spine (i.e., Cobb angle) [1–6]. While this outcome measure is important when comparing operative variables in spinal fusion procedures, the use of a global measurement such as Cobb angle may not be sensitive enough to detect the effects that operative variables, such as vertebral position within a tethering construct, may have on vertebral growth modulation in non-fusion procedures [7–9]. Attempts to study such variables in the clinical setting are complicated by the lack of access to direct specimens or resolution of indirect measurement techniques.

Historically, nearly all preclinical developments of vertebral body tethering (VBT) and other vertebral growth modulation studies have relied on use of the inverse approach [10–20] which inferred the effectiveness of a growth modulation technique by its ability to create a deformity, or a two-stepped approach [9, 21–26], in which a deformity was surgically created and then surgically corrected. These previous studies failed to use a natively deformed spine to study growth modulation techniques [27]. We have previously described a hyper-kyphotic porcine model, produced through maternal and neonatal vitamin D restriction in diets with surplus calcium and phosphorus [28], that exhibits a progressive kyphotic deformity during growth [28], resembling Scheuermann’s disease in humans [29–32]. These hyper-kyphotic swine can be predictably produced as offspring of conventional, crossbred dams fed hypovitaminosis D diets during gestation and lactation with additional modifications of diets fed to the offspring for a 3- to 4-week post-weaning phase [33]. The conventional crossbred genetic lines have well-characterized nutritional requirements with defined growth and reproductive developmental traits. We have employed the use of this non-surgically induced spinal deformity model to better understand deformity progression and growth modulation in the natively deformed spine. This novel, large animal model facilitates the investigation of surgical variables not easily or ethically testable in children.

While more popular as an alternative surgical treatment for scoliosis, vertebral growth modulation has been described in sagittal plane deformities. Lowe et al. demonstrated feasibility of the technique to decreased kyphosis and vertebral wedging in the ovine model [34]. Clinically, this technique was found successful in modulating vertebral growth in a young patient with kyphoscoliosis [35] and more recently an adaptation of the technique has been clinically described to correct Scheuermann’s kyphosis [36]. The purpose of this work was to use our hyper-kyphotic porcine model [28, 37] to directly assess the effect that vertebral position within a posterior VBT construct had on: mean and differential regional vertebral growth rates, the percent vertebral growth modulation, physeal and epiphyseal morphology, the relative location of the nucleus pulposis, and changes in vertebral shape as a result of tether mediated growth modulation.

## Methods

**Fig. 1:**
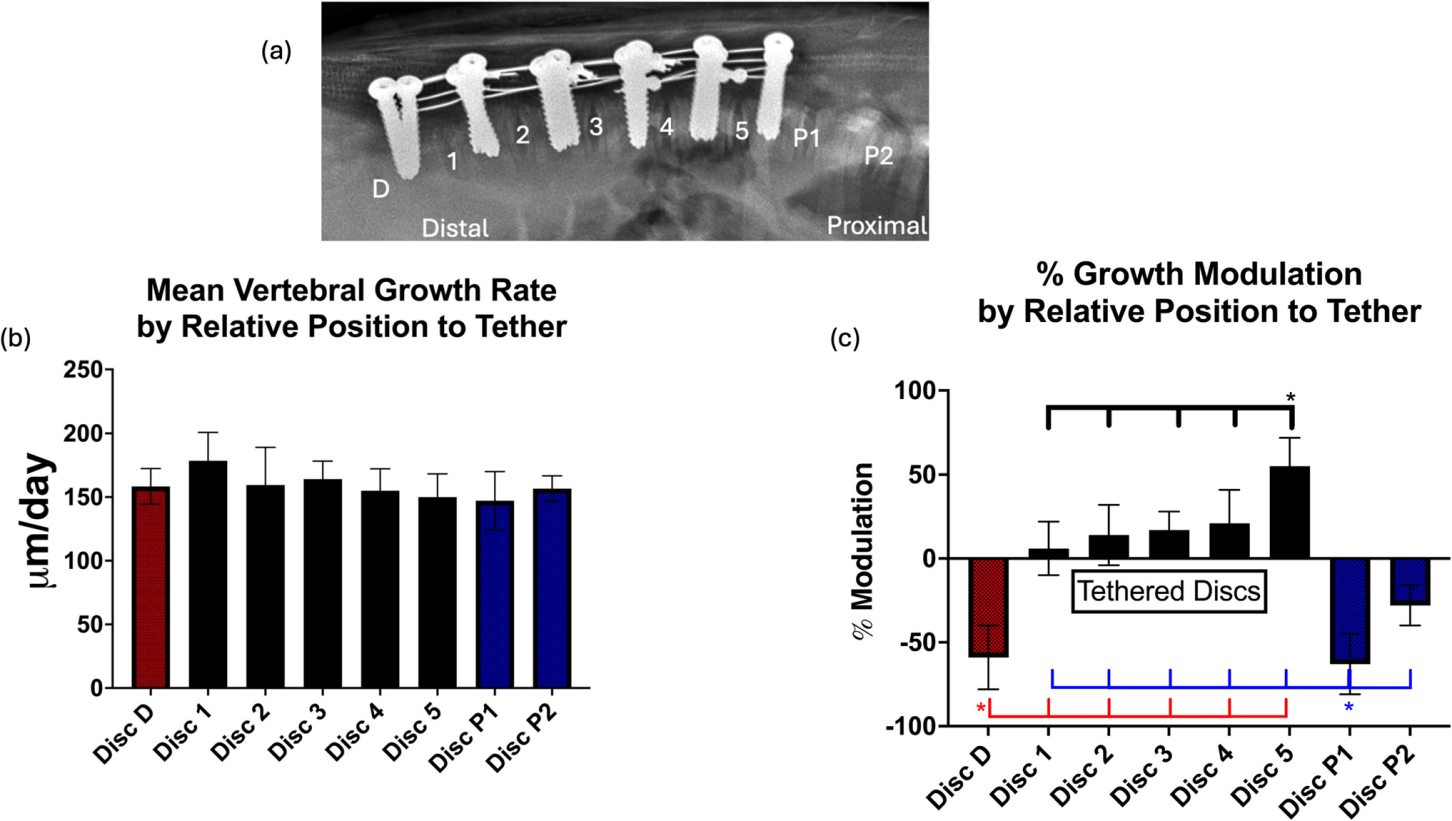
**a** Lateral radiograph highlighting the denotations used in this manuscript. **b** Mean vertebral growth rate as a sum of growth at each pair (proximal and distal) of vertebral physes within each disk level. **c** % Growth modulation at each level. Note the effectiveness of the tether to therapeutically modulate growth and the reversal of % growth modulation at the junctional kyphotic levels

Six, eight- to nine-week-old hyper-kyphotic swine [28], underwent multi-level posterior compressive tethering with high (*N* = 3, 25.6N) or low (*N* = 3, 5N) tension applied equally to each vertebral level of tethered. The apex of the deformity was confirmed on radiographs and Wiltse style approach was used. The articular process of the vertebra was palpated and soft tissue attachments sharply detached. A rongeur was used to nip off the posterior caudal corner under which a “blush” of vascular bone was observed. A cervical awl was then directed ~ 20 degrees caudal and about 20–30 degrees medial with tip pointed medially. The Lenke type awl was used and advanced between 20 and 30 mm in depth. A pedicle feeler was then used to sound medial, lateral, inferior, and superior and deep to ensure that the path was contained within the pedicle and the vertebral body. Guide pins for the cannulated screws were placed in all the tracts and lateral radiographs taken to confirm placement. Cannulated stainless steel fully threaded screws (5.5 × 28–32 mm) (OrthoPediatrics (Warsaw, IN) were then placed over the K wires and advanced into the pedicles and vertebral body until felt as if they had inserted into the ventral cortical bone of the vertebral body, or as far as they could be inserted to leave room for the tether cable to pass between the screw head and lamina of the vertebra. Screw placement was confirmed with lateral radiographs. Six pairs of cannulated screws were placed bilaterally into the pedicle of six contiguous vertebral levels. Five pairs of stainless steel cables (Atlas Cables, Medtronic, Minneapolis MN) were then placed spanning the five intervertebral disks in each animal. Cables were placed around each pair of screws of the cranial vertebra and caudal vertebra on each side. Starting at the apical pair of screws, a provisional tension holder was slid over the end of the wire on each side. The right side was tightened to the desired tension using handheld load cell (Nidek; Gamagori, Aichi, Japan) and then provisionally clamped. The left side was tightened in the same manner clamped, then permanently crimped. The provisional on the right side was released and the loop once again tightened to the desired tension, provisionally clamped, and crimped. The excess wire extending out of the loops cut and removed. This was done pairwise, starting at the apex, and then working distal and then proximal in an inside (apical) to outside manner, until all cables had been tensioned and crimped. Animals were recovered and received fluorochrome labels at two and four weeks post-operatively. Swine were euthanized at four weeks (~ 13 weeks of age) and tissues processed. As swine may have different numbers of vertebrae, for the purposes of this study, we defined the most distal instrumented disk space as D1 and the most proximal instrumented disk space as D5, adjacent uninstrumented disk spaces were labeled Dd (distally) and P1 and P2 (proximally) (Fig. 1a).

### Pulsed fluorochrome labeling, regional growth rate measurements, and growth modulation

**Fig. 2:**
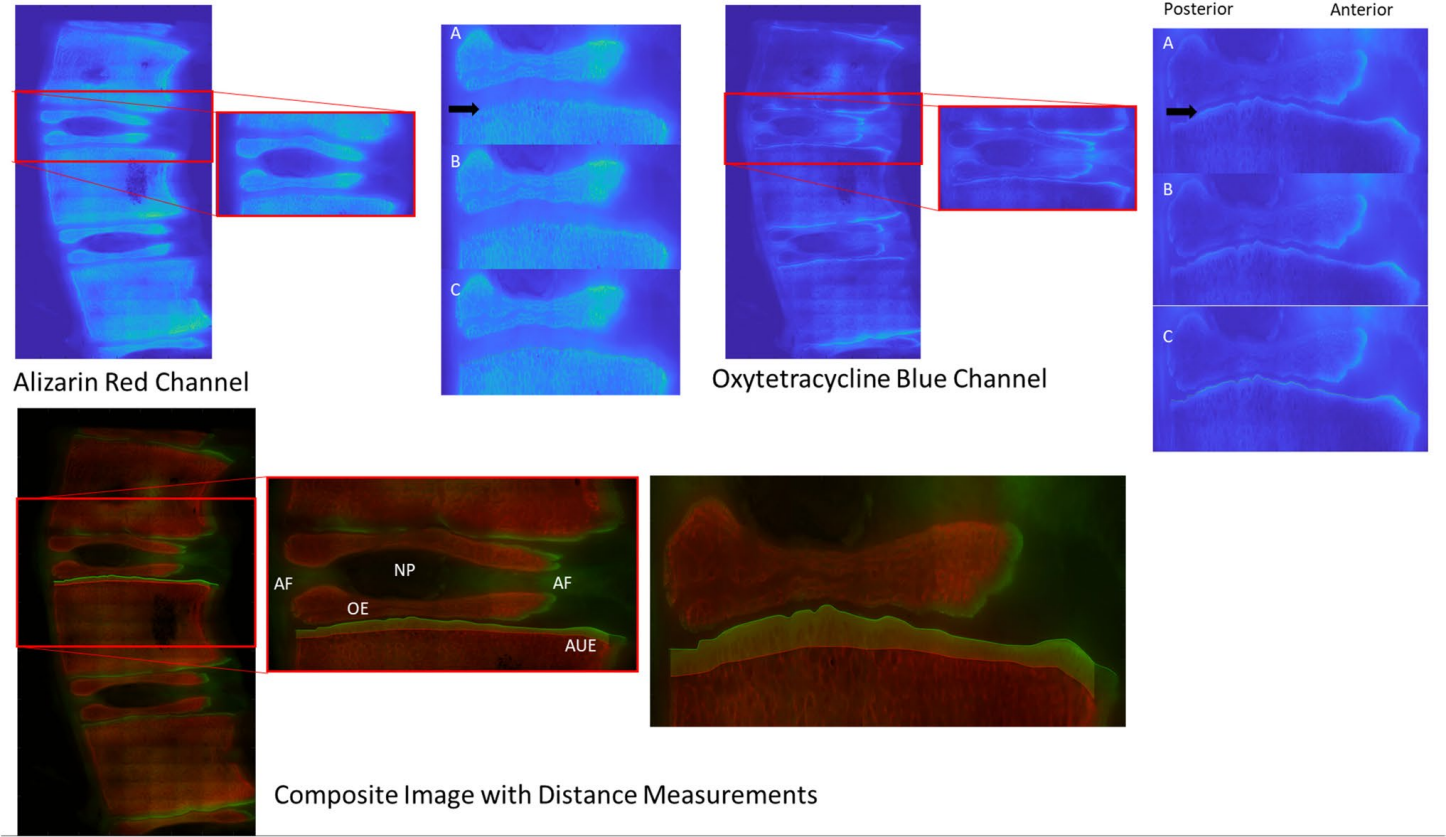
Step-by-step demonstration of growth rate measurements using pulsed fluorochrome labeling, example using a junctional kyphotic level outside the construct. (Top Left) Alizarin channel images are obtained and physis of interest selected. Note the arrow clearly indicating the alizarin front (**A**). Points are selected along this front following its contours (**B**). The Matlab program connects these points (**C**). Next (Top Right) the oxytetracycline channel image is evaluated. Note the arrow clearly indicating the oxytetracycline label near the chondrosseous junction (**A**). Points are selected along this front following its contours (**B**). The Matlab program connects these points (**C**). (Bottom set of Images) The composite image is produced, and the Matlab program measures the pixel-by-pixel distance measurements between the two fluorochrome label lines along the axis of longitudinal growth. This can be done for the entire growth plate or regionally as desired. Note the clear delineation between the annulus fibrosis (AF), nucleus pulposis (NP), ossified (OE) and unossified anterior epiphysis (UAE), and physis (located between the epiphysis and the remainder of the vertebral body). Notice the increased posterior > anterior growth in the junctional kyphotic level shown

Pulsed fluorochrome labeling was performed by administering Alizarin Red 12–14 days prior to harvest and Oxytetracycline on the day of harvest. Vertebrae were coronally sectioned into ~ 3 mm thick slabs utilizing an Isomet Precision saw (Buehler Isomet 2000; Lake Bluff, IL), and the entire spinal segment was visualized using a Nikon NiE upright microscope (Nikon Instruments; Melville, NY) set-up for epifluorescence at 4X magnification. A single central vertebral slab was evaluated for regional growth. Regional growth rate (µm/day) measurements were performed by measuring the distance between fluorochrome labels using a custom validated image analysis program [38] and dividing this distance by the time between fluorochrome label administration. The step-by-step process is demonstrated in Fig. 2. Growth rates were reported for the entire physis, anterior ¼, middle ½, and posterior ¼ of the vertebrae, as well as the rate of growth under the unossified anterior epiphysis. This regional division was based on the anatomic structure of the disk/epiphyseal structure and the different biomechanical properties annulus and nucleus pulposis (NP) [39–45]. As the annulus of the disk attaches to the anterior and posterior ~ 1/4 of the vertebra and the nucleus pulposis occupies the central ~ 1/2 of the disk space, the authors felt that the different mechanical properties of the annulus and NP would transmit tether forces differently to each region of the adjacent growth plates. In this manuscript, *we have defined growth modulation* as *% Growth Modulation*, defined as:

$$\frac{{{\mathrm{Anterior}} \frac{1}{4} {\mathrm{growth}}\;{\mathrm{rate}} - {\mathrm{Posterior}} \frac{1}{4} {\mathrm{growth}}\;{\mathrm{rate}}}}{{{\mathrm{Total}}\;{\mathrm{mean}}\;{\mathrm{vertebral}}\;{\mathrm{growth}}\;rate}} \times 100$$

This equation evaluates the differences in growth rates (anterior versus posterior) and normalizes the differences by the overall vertebral physeal growth rates to account for different rates of growth in individual animals. Thus, in our kyphotic model, a positive % Growth Modulation is indicative of (therapeutic) growth modulation (anterior > posterior growth), whereas negative % growth modulation is indicative of worsening kyphosis. Depending on the analysis, the proximal and distal physes within each disk space were analyzed separately to evaluate differential (proximal versus distal) physeal response or combined (the sum of the distal physeal growth of the proximal vertebra and the proximal physeal growth rate of the distal vertebra) to evaluate the overall effect of location within the tethering construct at each disk space (i.e., disk 1, D1 = most distal instrumented disk, disk 5, D5 = most proximal instrumented disk).

### Physeal Thickness

**Fig. 3:**
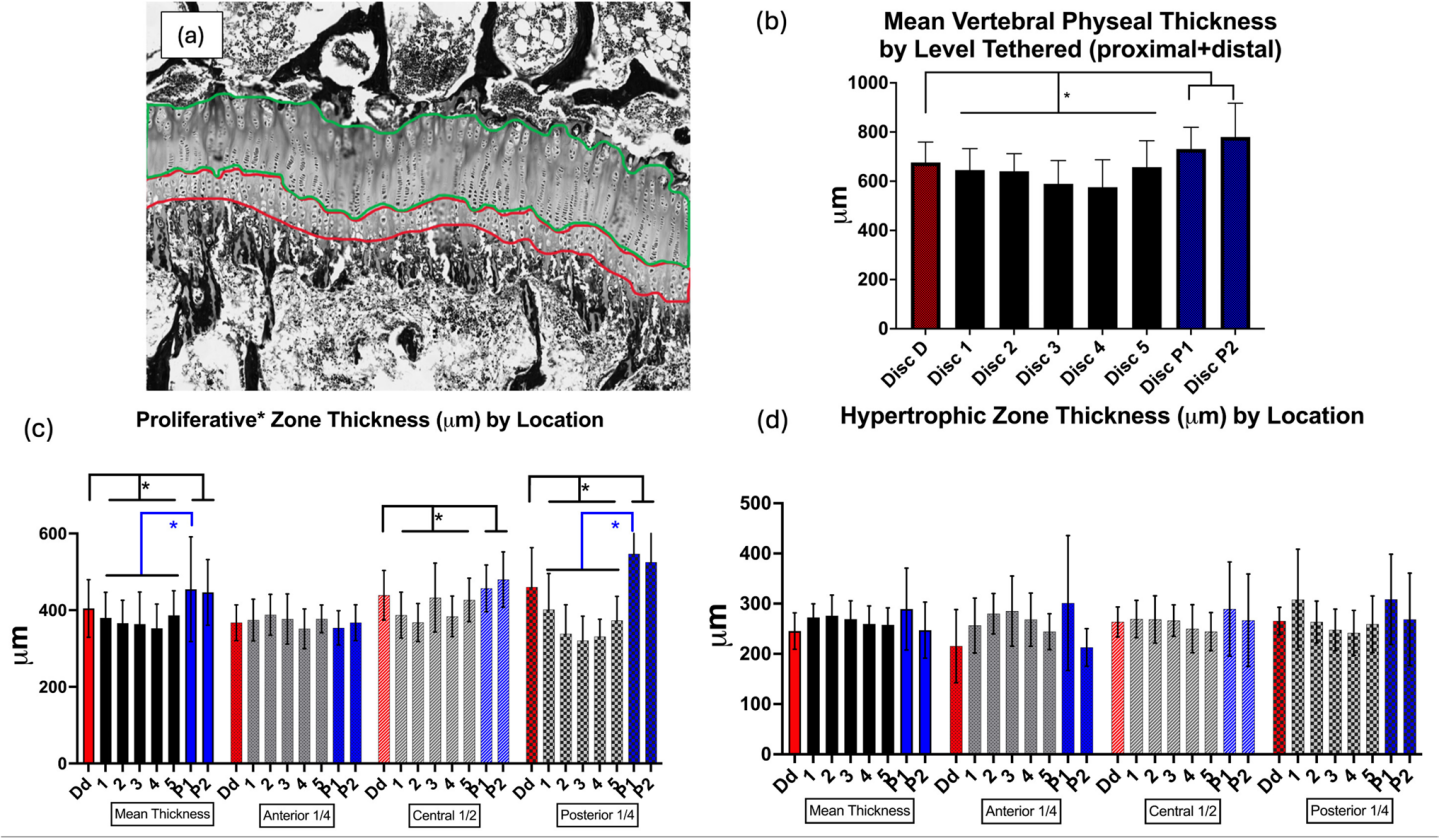
**a** Digitized micrograph demonstrating the zones of the vertebral physis analyzed **b** Mean vertebral physeal thickness (proximal physis + distal physis) by relative location to multi-tether construct. **c** Overall proliferative and reserve zone thickness by disk level (left) and sagittal region: anterior ¼, middle ½, and posterior ¼ on the vertebra. **d** Overall hypertrophic zone thickness by disk level (left) and sagittal region: anterior ¼, middle ½, and posterior ¼ on the vertebra. Despite similar growth rates, notice the centrally tethered physes appear thinner in the multi-tether construct. The reserve/proliferative zone appears most affected by compression as thinning of this zone is most seen in the central and posterior tethered physes compared to adjacent uninstrumented levels. Conversely, the hypertrophic zone appears taller anteriorly in the region where the spine is unloaded

Additional central midsagittal plane slabs taken from the vertebral segments were processed for histology  ~ 4 mm samples were fixed in 10% neutral buffered formalin (NBF) for ~ 1 week and decalcified using 15% ethylenediaminetetraacetic acid (EDTA) for ~ 14 days. Completed decalcification was assessed using Faxitron UltraFocusDXA, in radiographic mode. Decalcified sections were submitted to the Translational Research Initiatives in Pathology (TRIP) laboratories (UW-WIMR) for paraffin embedding, sectioning, and staining with Hematoxylin and Eosin and Masson’s Trichrome. Slides were imaged using the Aperio AT2 at 0.5 µm/pixel resolution at 20 × magnification. Scanned images (.svs) were then analyzed using BioQuant software (BioQuant Image Analysis Corporation, Nashville, TN). In BioQuant Software, an area was drawn and then calculated based on user specifications. The specification of interest was the average thickness of any given area (i.e., Hypertrophic, proliferative, reserve zone, etc. (Fig. 3a). The summation of the hypertrophic zone and proliferative zone was the focus and what composed of the 'total thickness' (area of focus). Red Area = Hypertrophic Zone, Green Area = Proliferative Zone.

### Epiphyseal ossification

**Fig. 4:**
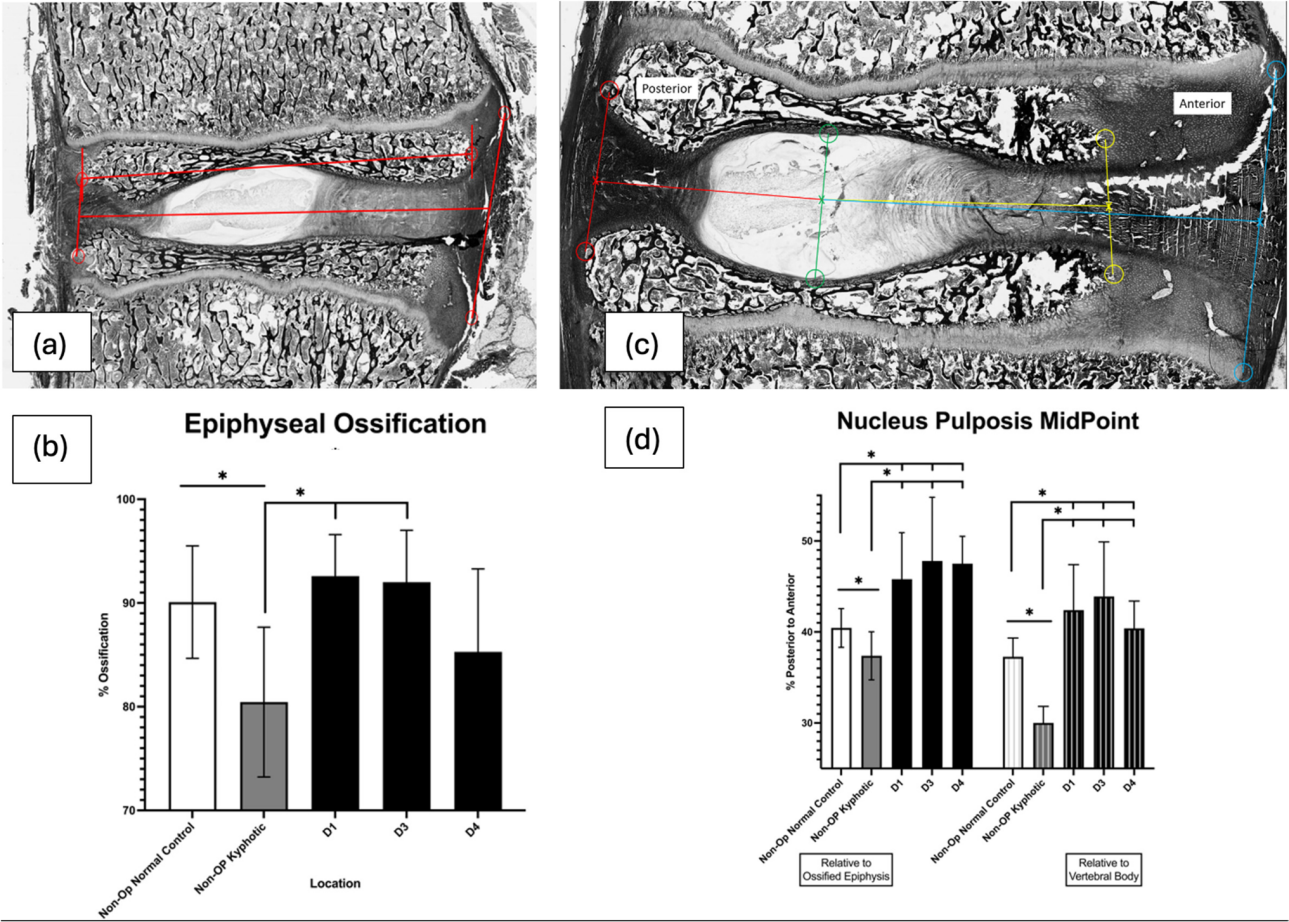
**a** Image of single vertebral endplate with ossified and unossified regions of the epiphysis. **b** Decreased (anterior) vertebral epiphyseal ossification is seen in non-operative kyphotic swine (gray), this differs from normal, non-kyphotic swine(white), increased anterior epiphyseal ossification was found at the tethered disk spaces (black). **c** Image demonstrating the measurements made determining the sagittal midpoint location of the NP (green), relative to the ossified epiphysis (yellow) or the entire vertebral body (red to blue) **d** The center of the (NP) was positioned more anterior on the epiphysis and vertebral body in the normal non-kyphotic swine than in the non-operative kyphotic swine. The NP shifts further anterior in the tethered levels (black)

The percentage of epiphyseal ossification was measured at each disk space using the scanned micrograph images and Bioquant software. The distance (A/P) of the ossified epiphysis was divided by the total distance (A/P) of the entire epiphysis including the unossified, cartilaginous regions for each epiphysis and multiplied by 100. The mean epiphyseal ossification for each disk level was then calculated from the proximal and distal epiphysis at each disk space (Fig. 4a).

### Nucleus pulposis midpoint location

The location of the sagittal geometric center of the nucleus pulposis (NP), (green line, Fig. 4c) was defined using the Bioquant software, at all available levels. The distance that the geometric NP center was located relative to the posterior to anterior edges of the ossified epiphysis or the overall vertebra. These were recorded as a percentage, posterior to anterior of the ossified epiphysis or the overall vertebra (i.e., 50% would indicate the geometric NP center was at the midpoint of the ossified epiphysis or vertebral body, < 50% would indicate a posterior position, and > 50% anterior).

### Additional non-operative controls

The epiphyseal ossification and location of nucleus pulposis midpoint location were also analyzed from the apical segments (three vertebral segments) of five additional ~ 13-week-old non-operative kyphotic and five normal control swine.

**Junctional kyphosis** To quantify junctional kyphosis, lateral digital radiographs (XJet, C.F.D. Devices, Scanzorosciate, Italy) at the time of harvest was performed. Sagittal Cobb angles encompassing the last tethered disk and the first untethered disk (Sante dicom viewer (Santesoft, Nicosia, Cypress), both proximally and distally were measured. Growth rates, physeal thickness, epiphyseal ossification, regional growth rates, and % growth modulation between the instrumented and uninstrumented levels were then made.

**CT scan and reconstruction** Following euthanasia and prior to processing the vertebra for growth rate analysis, sections of the spinal column ~ 10 vertebra encompassing the instrumented segments were resected *en bloc* and screws and cables removed. CT scan was then performed on a GE Medical Systems Discovery CT750 HD clinical CT scanner. (DICOM Viewer—RadiAnt; Poznan, Poland) RadiAnt software was used to create MPR images. Mid-sagittal images were then used to measure the individual vertebral Cobb angles. Kyphotic angles were denoted positive, and negative as lordotic. The Cobb angle for each vertebra based on position within the multi-tether construct, (vertebra 1 (V1) the most distal, vertebra 6 (V6) the most proximal). Mean sagittal vertebral shape was then assessed based on position within the construct.

**Outlier testing** To evaluate the effect of samples with instrumentation failure, potential outliers among the six samples were evaluated using Grubbs' test at a significance level of *a* = 0.05. Each sample that had a failure was designated a 'fail' for this calculation. The Grubbs' statistics (*G*) was calculated as the maximum absolute deviation from the sample mean, normalized by the sample standard deviation (*G* = 1.59). The calculated *G* was compared against the critical value (G Crit) derived from the *t* distribution with *n*-2 degrees of freedom (*G* Crit = 1.93). If *G* > *G* Crit, the corresponding sample was considered a statistically significant outlier.

### Statistical analysis

Vertebral Growth rates, % growth modulation, physeal thickness, epiphyseal ossification, and NP location were described with means and STD. ANOVA was performed comparing the % epiphyseal ossification and NP location between controls and the tethered vertebrae with a p value of 0.05. Given the number of analyses performed from some of these data sets a Bonferroni correction was performed to determine significance, with kappa values ranging from 7 to 13 depending on data leading to an adjusted alpha of 0.004–0.008 for these analyses.

One-tail t tests were conducted comparing any given vertebra against all other individual vertebra for both low- and high-tension tethering. Complete sub-analyses of the data based on tether tension (high vs. low) can be found in the supplemental materials.

## Results

**Mean vertebral growth rates and % growth modulation by relative position in posterior tether construct** Mean growth rates decrease from distal (lower lumbar) to proximal (T/L junction); however, this did not reach statistical significance (Fig. 1b). Surprisingly, the mean physeal growth rates of the tethered disk spaces (disks 1–5), 164 ± 20um/day were not less than adjacent untethered disks, 154 ± 16. As expected, mean growth modulation was significantly greater for the tethered versus non-tethered disk spaces (*p* < 0.0001, adj *p* = 0.004). Growth modulation appeared progressively greater the more proximal the tethered disk space; however, only the most proximal instrumented vertebral level (disk 5) was found to have a greater % growth modulation (55 ± 17%) than any other levels with ranges of 7%–21% at the other 4 disks within the construct (*p* = 0.0001, adj p value 0.004) (Fig. 1c). Disk 5 corresponded anatomically to the 2nd and 3rd most distal thoracic levels. No significant % modulation differences were found between other tethered anatomic levels or based on initial tension applied (see Supplemental Materials). Junctional kyphosis was observed through the significant reversal (negative) of growth rates modulation at the adjacent, uninstrumented vertebral levels (*p* < 0.0001, adj p value 0.004).

**Differences in physeal structure by relative location within posterior tethering construct** Tethering caused the physis to become thinner when compared to the adjacent levels (621 ± 36 vs 728 ± 52 um; *p* = 0.001, adj *p* = 0.004) (Fig. 3b). The proliferative/reserve zones made up the majority of the vertebral physeal height. Tethering had the greatest effect on thickness of the proliferative/reserve zone as this region was significantly thinner at tethered levels (367 ± 68 vs 435 ± 99, *p* = 00005, adj *p* = 0.004), in the central and posterior zones of the vertebrae under the compression of the posterior tether (Fig. 3c). The hypertrophic zone thickness was less affected by the tethering as no significant differences were found (Fig. 3d).

### Epiphyseal ossification and center of NP location by relative position to construct

**Fig. 5:**
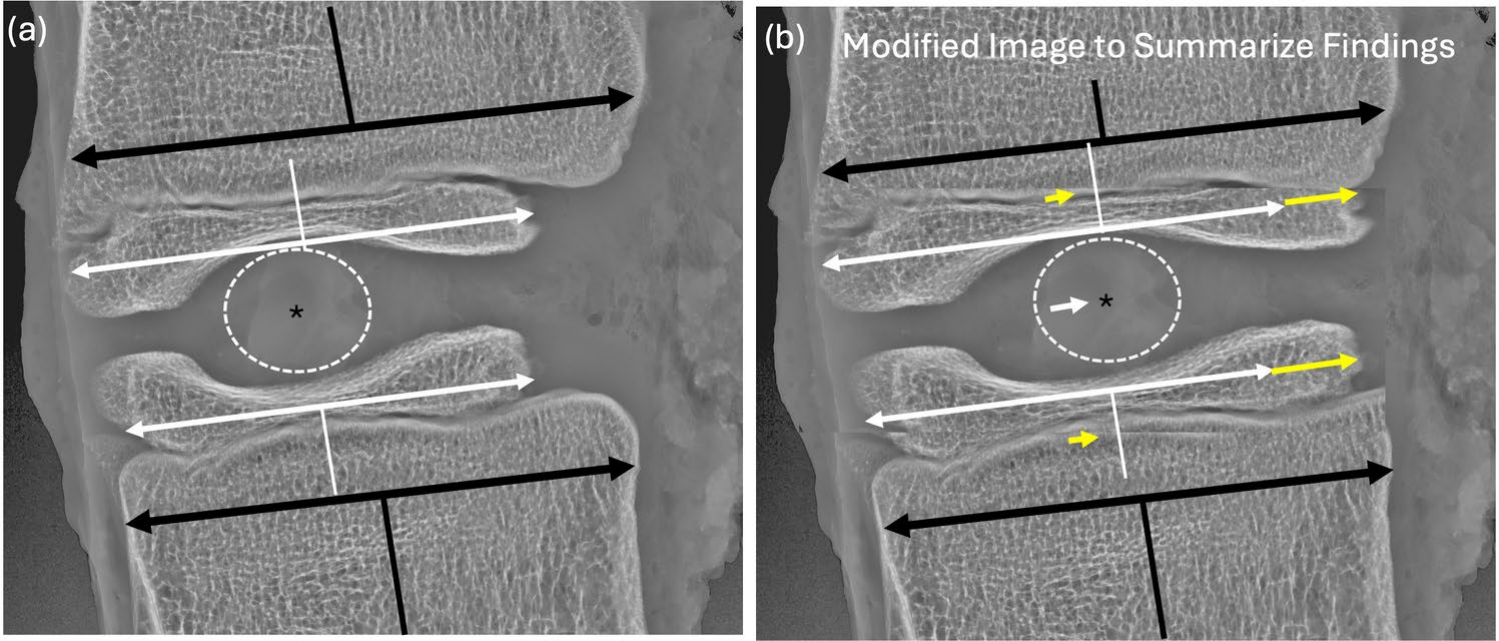
Modified radiographic images to summarize the interactions between NP and epiphyseal ossification. In the kyphotic spine the center of the ossified epiphysis (white vertical lines and double arrows) is positioned posterior to the center of the vertebral body (Black vertical lines and double arrows) while the center of the NP (asterisk) sits posterior to posterior to the center of the ossified epiphysis and therefore, very posterior to the center of the vertebral body. Tethering appears to shift the center of the NP toward the center of the ossified epiphysis (small single white arrow), while this is occurring the anterior epiphysis is also ossifying (yellow arrows), thus the NP is also shifting toward the center of the vertebral body. This suggests that in addition to correcting spinal deformity by the altering vertebral body shape through growth modulation at the physis, structural changes are also occurring in the epiphysis following pVBT

In the non-operative kyphotic swine, a larger portion (19.6 ± 0.1% vs 9.9 ± 0.1%, *p* < 0.001) of the anterior vertebral epiphysis was unossified when compared with non-operative, non-kyphotic controls. The midpoint of the nucleus pulposis was positioned further posterior on the vertebral epiphysis in the kyphotic swine (37.4 ± 0.04% vs 40.4 ± 0.03% *p* = 0.02) and more posterior on the overall vertebral body (30.0 ± 0.04% vs 37.3 ± 0.03% *p* < 0.001) than in the non-operative control animals. Tethering significantly increased the anterior ossification of the epiphysis (Fig. 4a, b) and shifted the nucleus pulposis significantly more anterior on the epiphysis and vertebral body than both non-operative controls (Fig. 4c, d). A schematic from modified radiographs summarizing these findings can be found in Fig. 5.

### Comparison of the mean vertebral growth rates and % growth modulation between the proximal and distal physes within each tethered disk space

**Fig. 6:**
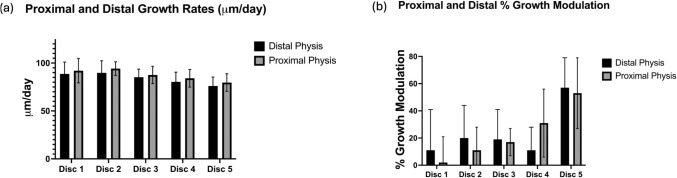
**a** Comparison of mean proximal and distal vertebral physeal growth rates at each disk level. **b** Comparison of % growth modulation of the proximal and distal vertebral physis within each disk. Note growth rates and % modulation is similar within each disk pair, however growth rates become slower the more proximal in the construct, whereas % modulation increases

Within each disk space, the mean proximal physeal growth rate was not significantly different than the mean distal physeal growth rate at that level (Fig. 6a). Similarly, no significant difference was found in the % growth modulation between the proximal and distal physes within each pair (Fig. 6b).

### Sagittal vertebral shape differed by position within construct

**Fig. 7:**
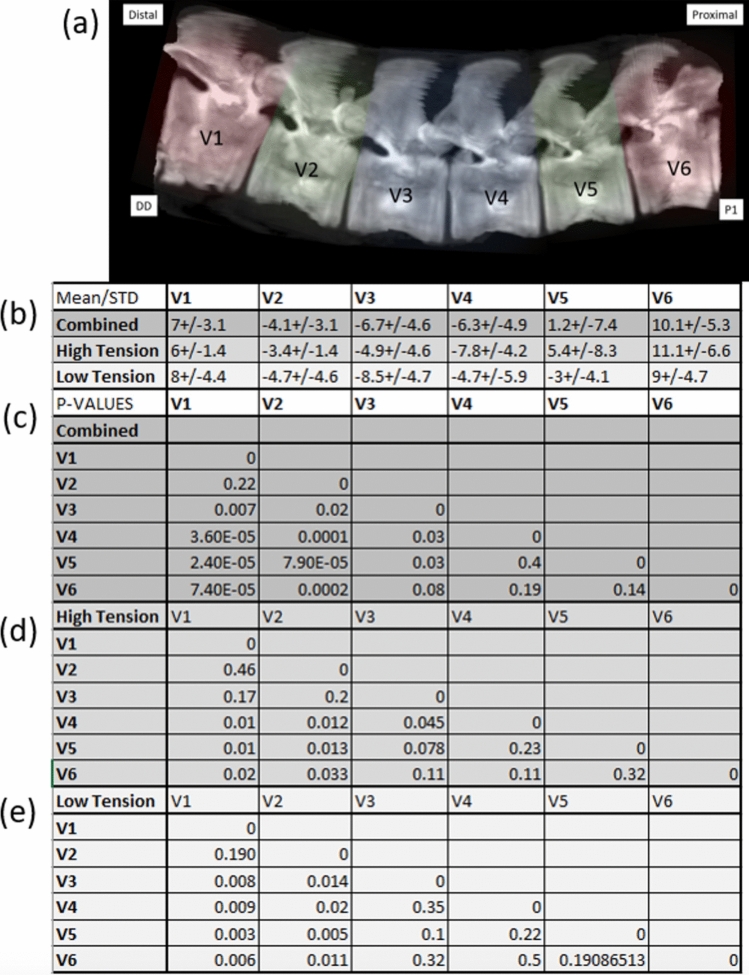
**a** Denotation of vertebral levels within the multi-tether construct shaded to represent the central/apical pair (blue), the end vertebra (pink), and the intermediate vertebra (green); 3D reconstruction of sample following implant removal. **b** The mean Cobb angle and STD of each level in the overall analysis and by high- and low-tension cohorts. **c**–**e** pairwise p values comparing mean Cobb angles for each vertebral position within the construct (**c** overall, **d** high tension only, **e** low tension only)

Overall, the central pair of the vertebra demonstrated increased lordosis, V3 −6.3 ± 4.9° and V4 −6.7 ± 4.6°, while the end vertebrae were kyphotic, V1 10.1 ± 5.3° and V6 7 ± 3.1°. The Cobb angles of the instrumented vertebra, V2 and V5, were intermediate of the central and outer vertebra. Mean Cobb angles and pairwise comparisons are shown in Fig. 7. As overall Cobb angle correction was found to be different between high- and low-tension constructs in our accompanying manuscript, separate analyses are also presented, but similar overall trends still observed.

### Junctional kyphosis: macroscale Cobb angle and microscale growth modulation

**Fig. 8:**
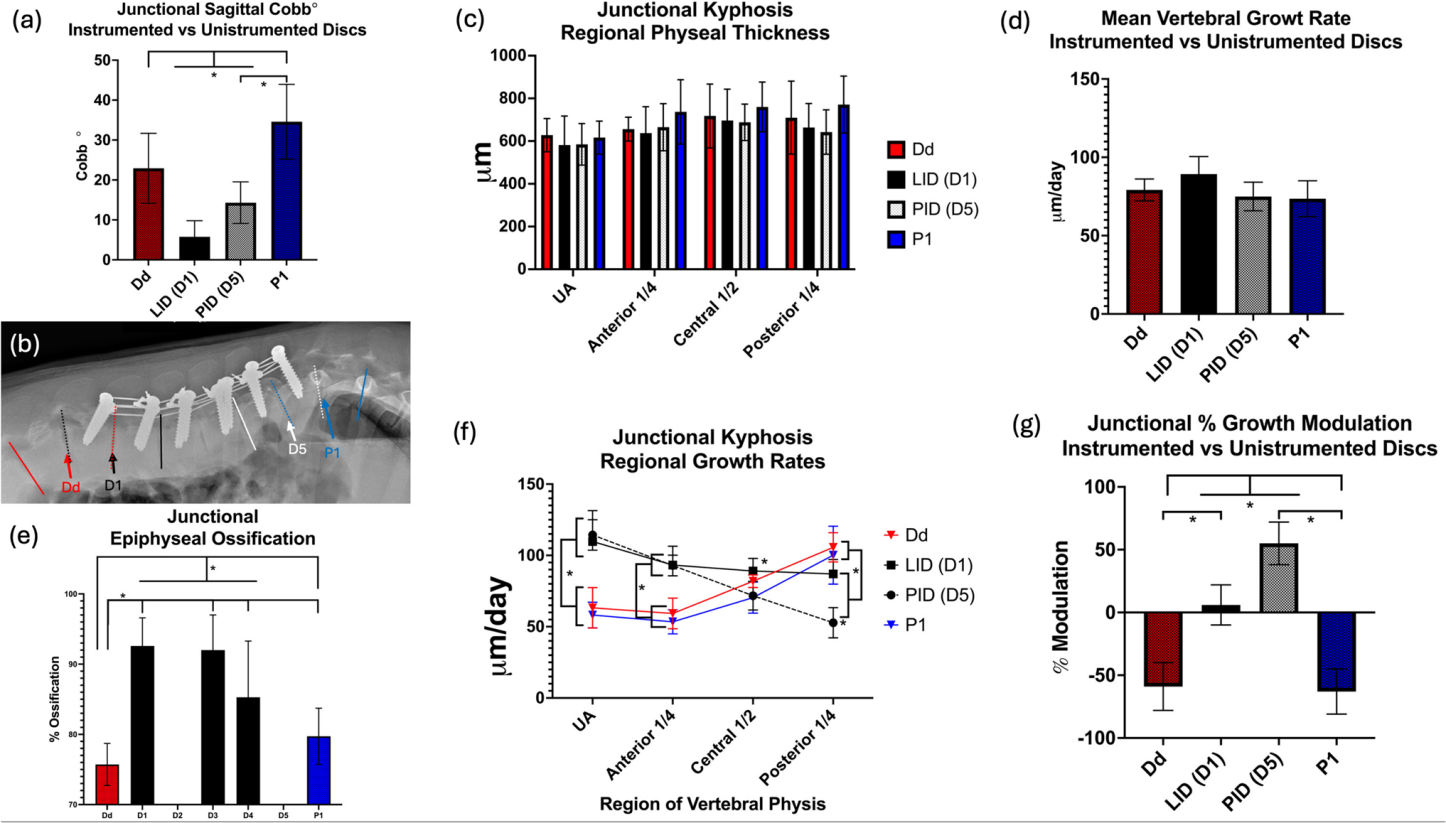
**a** The macroscopic effects of junctional kyphosis as measured by mean sagittal Cobb angle between the first distal (Dd) and proximal (P1) uninstrumented disk space and the Lowest Instrumented Disk (LID, D1) and the Proximal Instrumented Disk (PID). **b** Lateral radiograph of severe case of junctional kyphosis demonstrating measurements made. Junctional kyphosis did not cause any difference in regional physeal thicknesses (**c**) or growth rates (**d**). However, the epiphysis at the un-instrumented adjacent levels were significantly less ossified anteriorly than the instrumented levels, (**e**), the regional growth distribution inverted (**f**), thus leading to a reversal of % growth modulation (**g**)

Increased overall kyphotic Cobb angle was found between at the disks outside the tethering construct versus the last instrumented disk space (*p* = 0.00005, adj *p* = 0.006), Fig. 8a. A severe example of junctional kyphosis is demonstrated in Fig. 8b. No significant differences were found in the physeal thickness or mean growth rates between the end-instrumented disk spaces and the adjacent uninstrumented (Fig. 8c, d) However, decreased epiphyseal ossification was found at the uninstrumented levels compared with the instrumented levels (Fig. 8e). While no significant differences in mean vertebral growth rates were observed between the instrumented and uninstrumented levels, the regional distribution of that growth was significantly different as the instrumented levels demonstrated greater anterior and less posterior growth (*p* < 0.0001, adj *p* = 0.007) than the adjacent uninstrumented levels resulting in opposite and significant vertebral growth modulation (*p* < 0.0001, adj *p* = 0.006), (Fig. 8f, g).

### Evaluation of implant complications on growth modulation and physeal thickness

**Fig. 9:**
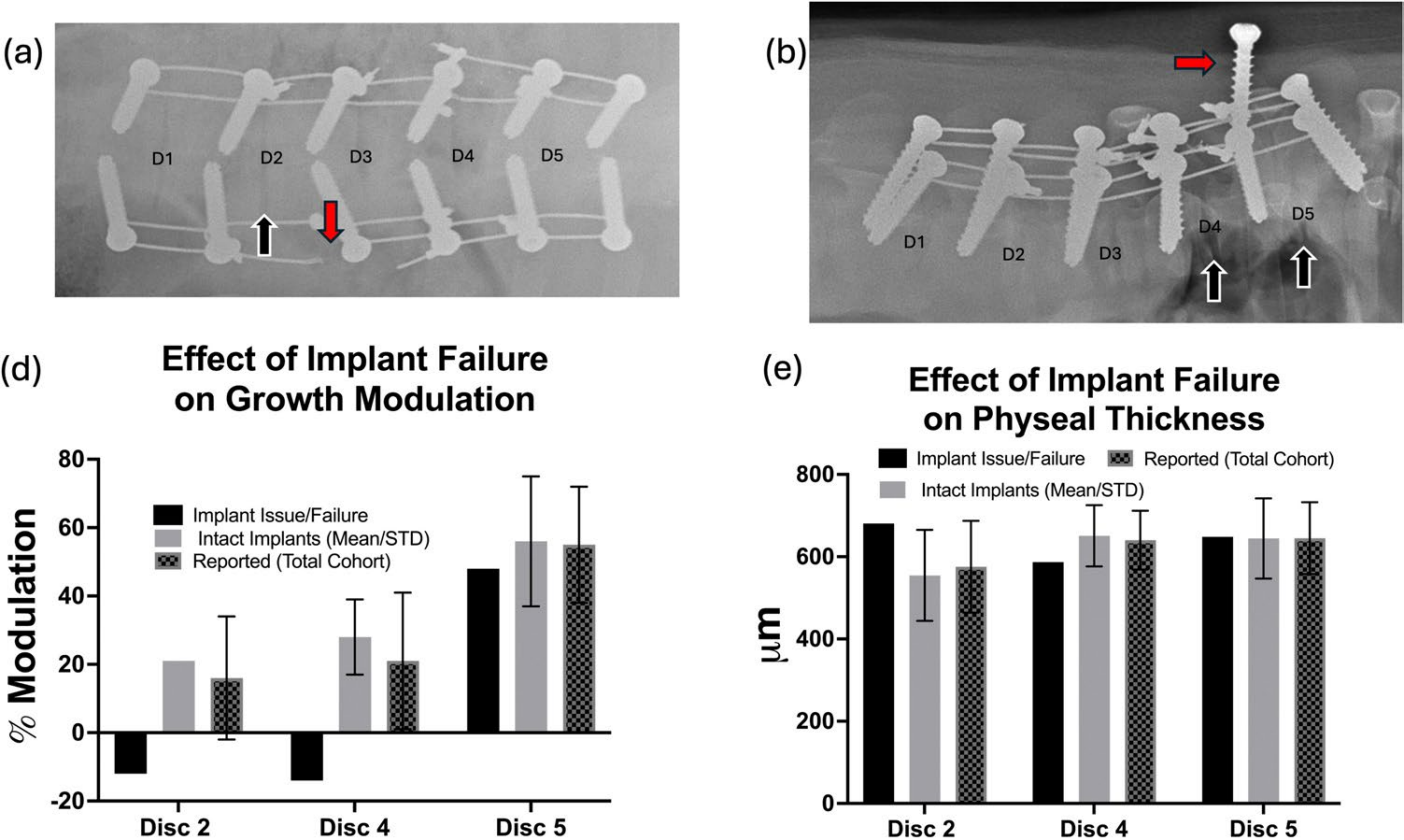
**a** AP radiograph demonstrating the single broken tether (red arrow) and the potentially affected disk space D2 (black arrow). **b** Lateral radiograph showing screw backing out (red arrow). As two cables are wrapped around the screw, two potentially tethered spaces could be affected, D4 and D5, (black arrows). **c** graphical comparison of growth modulation at the potentially affected disk spaces. Most of the effect was observed at D2 and D4, these may have lessened the overall modulation measured at those levels. **d** implant failure had no clear effect on physeal thickness

Two of the six animals had implant issues identified on final radiographs (Fig. 9). One animal had a unilateral failure of one of the second most distal tethers (Fig. 9a), while the other had a rather unusual unilateral screw backing out at the second most proximal level. These implant complications may have led to altered growth modulation at three potential disk spaces. To evaluate this, we compared the mean growth modulation and physeal thickness data at each of these disk spaces, excluding the potentially affected data. These results are shown in Fig. 9d, e. Formal statistical outlier testing, i.e., Grubb’s test did not identify either of the two failed samples as statistical outliers (*G* <  = *G* Crit). This indicates that, at the 0.05 significance level, there was insufficient evidence to conclude that the failed samples were inconsistent with the rest of the dataset. Nevertheless, sensitivity analysis was performed by recalculating descriptive statistics with and without the failed samples to assess their potential influence on the overall outcome.

## Discussion

Applying micro-scale measurement techniques to a vertebral level-by-level and physis-by-physis approach, allowed in-depth characterization of the vertebral physes within the multi-level tether construct. This approach demonstrated that compressive tethering in a young, flexible, spine did not significantly inhibit mean vertebral growth. Using these techniques demonstrated that the proximal and distal physes within a given tethered disk space responded similarly to the tethering, whether the same would hold true in the upright human spine with the additional effects of gravity remains unknown. Furthermore, despite being placed under similar tension, the most proximal tethered disk level in our constructs experienced the most growth modulation during the period tested. This anatomic level corresponded to the second to third most distal thoracic disk space. The tethered physes had narrower proliferative/reserve zones and this finding was most pronounced in the middle and posterior vertebral regions, indicating that this zone may be sensitive to compression due to the presence of the posterior tethers. Stokes et al. noted that the proliferative and hypertrophic zones in the rat caudal vertebra decreased with static compressive load and increased with distraction [46]; whereas cyclical loading [47–50] has been demonstrated to increase hypertrophic zone height. As clinicians are more accustomed to thinking about spinal deformities in terms of Cobb angle, it is important to note that this study did not focus on Cobb angle as this measurement is the sum of disk flexibility, vertebral deformity, tether tension, and *vertebral growth modulation,* but rather we used *% growth modulation* as the primary outcome measure. This new measurement allows a unique insight into the true, tether induced asymmetric vertebral growth that is occurring, independent of initial vertebral deformity or disk mobility. As this measure is normalized to the mean vertebral growth, it allows comparisons of the effects of the tether at different locations.

A unique characteristic in our hyper-kyphotic swine model is the unossified anterior vertebral epiphysis [28]. As anterior epiphyseal ossification was normalized at several of the instrumented levels, our findings suggest this is lack of normal ossification is due to the mechanical load placed on the anterior vertebrae in the kyphotic spine and that it can be corrected if the mechanical environment is improved, reminiscent of the proximal tibia in infantile tibia vara [51]. In addition to the anterior ossification, the epiphysis also appears to remodel in response to the nucleus pulposis as it shifts forward in response to the tethering (Fig. 5). As the human vertebral epiphysis is unossified, these changes are not typically visualized in clinical cases. Unlike previous reports of anterior VBT [16], the nucleus pulposis translated anteriorly away from the tether as the deformity was corrected and over-corrected.

While bilateral multi-level tethers were centered over the apical kyphotic segments, the five tethered levels (six vertebrae), did not fully span the deformity to the first lordotic segment. As such, junctional kyphosis was observed proximally and distally. Junctional kyphosis is an unwanted secondary sagittal deformity that occurs at vertebral levels adjacent to spinal instrumentation. A recent systematic review in pediatric patients has identified that increasing number of distractions in EOS (early onset scoliosis), disruption of posterior elements, and greater pre-operative kyphosis (thoracic) and lordosis (lumbar), longer fusion constructs, posterior versus anterior instrumentation, and greater sagittal correction as risk factors for junctional kyphosis [52]. Outside of the baseline kyphosis in our swine and the use of posterior instrumentation, none of the other reported risk factors appear to be present in our model. In agreement with our findings, a previous case report using posterior tethering demonstrated junctional kyphosis [35]. While many clinicians ascribe PJK as a side effect of gravity on an instrumented spine, our results in the quadruped would suggest that the pathophysiology and biomechanics are not the effects of gravity alone. Similarly, implant rigidity [53–55] and posterior midline tissue disruption [52, 54] are commonly attributed to the development of junctional kyphosis; however, the Wiltse approach and the flexible cables were used in this study, again demonstrating that these factors alone are not responsible for junctional kyphosis. While our findings do not fully explain the pathophysiology underlying junctional kyphosis and do not recapitulate the effects of bipedal ambulation and gravity, they do shed new light on this unwanted phenomenon in the growing spine. First, our fluorochrome labeling revealed that the mean vertebral growth rates between the tethered and untethered vertebra at these junctional kyphotic segments are similar, meaning the tethering does not appear to significantly inhibit growth. However, quite strikingly in our data, growth modulation between tethered and untethered levels was in the complete opposite direction (Fig. 8). *This means that the junctional vertebral bodies were actively growing (deforming) to become more kyphotic (wedged).* This finding, which likely also occurs in at a slower pace in growing bipedal children but is much harder to accurately measure clinically, indicates that the growing spine is not a simple passive structure that is being posteriorly “stretched out” by the surrounding mechanical environment (muscles, gravity, implant stiffness). The physes of the uninstrumented adjacent vertebral levels are active participants in progression of junctional kyphosis that responds to the environment by actively growing more posteriorly than anteriorly, further worsening the vicious cycle. Strategies aimed at reversing this mechanical environment or eliminating growth (i.e., extending instrumentation to the first lordotic disk, bracing in a lordotic position, waiting until growth is completed) might be reasonable strategies to thwart its progression.

While the non-surgically induced spinal deformities in our swine are an improvement over modulating the growth of a straight spine, questions regarding the generalizability of using quadrupeds to study spine deformity and vertebral modulation arise. A review of large animal models currently available for the study of fusionless spinal deformity technologies included pigs, mini-pigs, lambs, and calves [27] all of which are quadrupeds. Surprisingly, while these quadrupeds do not completely mimic the axial loading that gravity exerts on the upright human spine, the rhythmic contractions of the longitudinal paraspinal muscles appear to exert as great or greater axial load than gravity does in the human [56]. Furthermore, growth study in long bones [57] has demonstrated that > 90% of growth occurs during recumbency. While this has not been proven in the spine, it may indicate that gravity may not have as great as an effect on deformity progression as one might surmise. While we cannot directly apply our findings to human kyphotic deformities, our data agree with previous clinical reports demonstrating clinical correction of human kyphotic deformities [34, 35] and the upright bipedal posture of humans may be more prone to junctional kyphosis [35].

This study has many limitations in addition to the use of a quadruped model as discussed above. Our sample size was limited to a sample of convenience from animals utilized in our previous report [58]. A post hoc power analysis based on D3 and D5% growth modulation (*N* = 6, α = 0.05, two-tailed) demonstrated power of 0.99 for the primary outcome measure of this study % growth modulation. Conversely, the sample size required (per group) to achieve 80% power for the effect size (*d* = 0.18) between D3 and D4, there would need to be 480 subjects per group to detect a difference at these levels. Despite our small sample size being prone to type II errors and possibly limiting the generalizability of our findings, we feel that the limited conclusions made from this restricted sample size are supported by the data. In attempts to maximize the samples available, the low- and high-tension cohorts from our previous study were grouped into the same analysis. Our previous report demonstrated that at the 2–4-week period, regional growth and growth modulation at the apical tethered level did not differ significantly [58]. To assess the potential confounding influence of tether tension, a separate analysis was performed using tether tension as a variable assessing final vertebral shape (Fig. 7). The effects of tether tension on all variables can be found in the supplemental materials. Outside the reported differences in the vertebral shape, no other significant differences were found between the cohorts (except that significance was lost in a few analyses, likely due to loss of sample size). Another limitation is the use of a single central vertebral slab to evaluate growth plate histology. While we believe for the kyphotic swine, the central sagittal slab is the most informative, slabs from other regions would have improved the studies rigor. The lack of pre-operative CT scans was another limitation due to the animal facilities where the animals having their procedure were housed not having a CT scanner and a policy that forbids them to return to the facility once they leave, thus CT imaging was limited to post euthanasia. The authors believe that the overall change in shape of the apical or centrally tethered vertebrae would have been even greater as these were often the most kyphotic wedged vertebrae preoperatively. As we lacked these pre-operative scans, we were limited in reporting only on the final vertebral shape. Another potentially perceived limitation is the short duration (4-week) follow-up. The authors however do not feel that this is a significant limitation for the purposes of this study, which were to evaluate the effects of vertebral tethering on vertebral growth modulation. Our findings demonstrate we were successful in doing that and final that vertebral shapes were different, suggesting that the vertebral shape did change over the short time. The authors would concede that this model, or at least the age of swine used in this study is not ideal for studying the long-term effects of the tether on disk health or implant fatigue failure. Despite the short follow-up, two of the six animals had implant issues on final radiographs. One had unilateral tether breakage at the second most distal instrumented level, whereas another had a unilateral screw back out at the second most proximal instrumented level. We have evaluated our data with and without the data from these failure levels, while the growth response does appear different at two of the three-potentially affected disk spaces (Fig. 9), the overall differences from these failures may have minimized the mean growth modulation reports and possibly obscured the trend of growth modulation increasing gradually from distal to proximal throughout the construct. Despite this potentially confounding factor, statistical outlier testing did not demonstrate these samples were outliers (Table 1) and we do not believe that our reported differences were significantly affected by these implant issues. Furthermore, while not a planned or desired result, these failures do provide a unique insight into what may be happening clinically to vertebral growth when a tether fails.

**Table 1:** Effect of failed samples (instrumentation failures) on descriptive statistics and outlier detection

Scenario	Mean + / − SD	*N*	Grubbs' test result
All SAMPLE (*n* = 6)	22.7% + / − 0.096	6	–
Excluding Fail 1	23.9% + / − 0.1	5	Not an outlier
Excluding Fail 2	25.8% + / − 0.07	5	Not an outlier
Excluding Both Fails	28% + / − 0.05	4	Neither an outlier

From this work, we have demonstrated that in the flexible kyphotic spine: growth modulation of the proximal and distal physis within a tethered level are similar, the mean growth rates of the tethered vertebra are similar to the adjacent untethered levels, the proliferative/reserve zones of the tethered physis are narrower in compressed regions (posterior) and growth modulation and ultimate vertebral shape may be different based on the location within a construct. This work also demonstrates asymmetric growth may contribute to junctional kyphosis. Future work will focus on understanding whether the observed differential growth modulation is anatomic level-related (distal thoracic spine) versus an effect of being proximally located within a pVBT construct and measuring the change in vertebral shape over time at each location.

## Supplementary Information

Below is the link to the electronic supplementary material.

Supplementary file1 (DOCX 275 KB)

## Data Availability

Data is available upon reasonable request.
