# Supplementary material for: Physeal effects of posterior VBT are not uniform throughout a multi-tether construct in the kyphotic swine model
Source: Spine Deform. 2025 Dec 19;14(2):335–48. doi: 10.1007/s43390-025-01247-0 (PMC12909388; doi:10.1007/s43390-025-01247-0)
Supplement: Supplementary file 1 [file 43390_2025_1247_MOESM1_ESM.docx]

Low Tension	1207-2	1213-2	1214-1					

Nucleus Pulposis Location

alpha 0.05; k 6; Reported p-values (both groups)

File: EXP 23101 Bioquant Data
Found in 'Low Tensions NP Location' tab

	%=distance Posterior VB to midpoint NP							% distance Posterior **ossified** epiphysis to midpoint NP						
	Mean	STD	P-value	Bonf α	Bonf sig	Dunn-Sidàk α	sig	Mean	STD	P-value	Bonf α	Bonf sig	Dunn-Sidàk α	sig
Mean % Across All Tethered	48.77%	3.91%	0.205	0.008333	no	0.008512445	no	42.26%	3.91%	0.571	0.008333	no	0.008512445	no
Mean % Across all Untethered	44.99%	3.83%	0.297	0.008333	no	0.008512445	no	36.67%	1.97%	0.181	0.008333	no	0.008512445	no
Mean %Across D and P1	45.66%	4.38%	0.561	0.008333	no	0.008512445	no	35.98%	1.96%	0.147	0.008333	no	0.008512445	no
Mean % Across P2	43.31%	2.66%	0.096	0.008333	no	0.008512445	no	36.75%	1.95%	0.801	0.008333	no	0.008512445	no
Between Tethered and Untethered			0.096	0.008333	no	0.008512445	no			0.801	0.008333	no	0.008512445	no
Mean % across Dd	48.33%	3.99%	0.186	0.008333	no	0.008512445	no	36.52%	2.45%	0.530	0.008333	no	0.008512445	no
Mean % Across D1	48.14%	3.17%	0.090	0.008333	no	0.008512445	no	43.18%	2.36%	0.588	0.008333	no	0.008512445	no
Mean % Across D2 (NA)														
Mean % Across D3	50.87%	5.49%	0.014	0.008333	no	0.008512445	no	44.58%	4.03%	0.072	0.008333	no	0.008512445	no
Mean % Across D4	47.30%	3.24%	0.848	0.008333	no	0.008512445	no	39.03%	3.76%	0.856	0.008333	no	0.008512445	no
Mean % Across D5 (NA)														
Mean % Across P1	42.99%	3.27%	0.064	0.008333	no	0.008512445	no	35.44%	1.64%	0.080	0.008333	no	0.008512445	no
Mean % Across P2	43.64%	2.58%	0.535	0.008333	no	0.008512445	no	38.05%	1.29%	0.151	0.008333	no	0.008512445	no

Epiphyseal Percent Ossification
Define

File: EXP 23101 Bioquant Data — alpha 0.05
Found in 'Low Tension %Epiphyseal Os — k 8 — Reported p-values

**% Epiphyseal Ossification**	Mean	STD	P-value	Bonf α	Bonf sig	Dunn-Sidàk α	sig
Mean % Across All Tethered	86.705%	5%	0.189	0.00625	no	0.006391151	no
Mean % Across all Untethered	81.831%	5%	0.002	0.00625	yes	0.006391151	yes
Mean %Across D and P1	79.096%	4%	0.015	0.00625	no	0.006391151	no
Mean % Across P2 & P1	84.936%	3%	0.090	0.00625	no	0.006391151	no
Between Tethered and Untethered			0.064	0.00625	no	0.006391151	no
Mean % across Dd	75.621%	1%	0.001	0.00625	yes	0.006391151	yes
Mean % Across D1	89.768%	2%	0.061	0.00625	no	0.006391151	no
Mean % Across D2 (NA)							
Mean % Across D3	87.771%	2%	0.250	0.00625	no	0.006391151	no
Mean % Across D4	82.577%	7%	0.585	0.00625	no	0.006391151	no
Mean % Across D5 (NA)							
Mean % Across P1	82.570%	3%	0.583	0.00625	no	0.006391151	no
Mean % Across P2	87.302%	3%	0.321	0.00625	no	0.006391151	no

Growth Plate Analysis

File: EXP 23101 Bioquant Data — alpha 0.05
Found in 'Low Tension GP Analysis' tab — k 13 — Reported p-values

	Mean Total Thickness	STD Total Thickness	p value	Bonf α	Bonf sig	Dunn-Sidàk α	sig	Mean Ant Unossified total Thickness	STD	p-value	Bonf α	Bonf sig	Dunn-Sidàk α	sig
Mean Total Thickness Tethered	627.170	97.241	0.829	0.003846	no	0.003937864	no	567.590	82.652	0.658	0.003846	no	0.003937864	no
Mean Total Thickness All Untethered	733.279	123.279	0.338	0.003846	no	0.003937864	no	631.538	70.128	0.175	0.003846	no	0.003937864	no
Mean Thickness D and P1	688.361	63.397	0.744	0.003846	no	0.003937864	no	618.494	83.044	0.168	0.003846	no	0.003937864	no
Mean Thickness P2 & P1	760.733	142.384	0.335	0.003846	no	0.003937864	no	613.678	76.549	0.181	0.003846	no	0.003937864	no
Between Tethered and Untethered			0.029	0.003846	no	0.003937864	no			0.066	0.003846	no	0.003937864	no
Mean Total Thickness Dd	678.370	57.654	0.862	0.003846	no	0.003937864	no	667.257	46.389	0.091	0.003846	no	0.003937864	no
Mean Total Thickness D1	665.015	137.238	0.976	0.003846	no	0.003937864	no	547.384	115.342	0.335	0.003846	no	0.003937864	no
Mean Total Thickness D2	581.697	118.819	0.185	0.003846	no	0.003937864	no	524.148	98.483	0.135	0.003846	no	0.003937864	no
Mean Total Thickness D3	597.779	135.519	0.285	0.003846	no	0.003937864	no	567.578	106.296	0.603	0.003846	no	0.003937864	no
Mean Total Thickness D4	628.963	71.758	0.561	0.003846	no	0.003937864	no	567.633	61.096	0.604	0.003846	no	0.003937864	no
Mean Total Thickness D5	662.395	36.215	0.945	0.003846	no	0.003937864	no	631.207	16.464	0.388	0.003846	no	0.003937864	no
Mean Total Thickness P1	698.352	80.152	0.631	0.003846	no	0.003937864	no	569.731	89.194	0.636	0.003846	no	0.003937864	no
Mean Total Thickness P2	823.114	180.513	0.010	0.003846	no	0.003937864	no	657.626	29.993	0.144	0.003846	no	0.003937864	no

	Mean Ant 1/4 Total Thickness	STD	p-value	Bonf α	Bonf sig	Dunn-Sidàk α	sig	Mean middle 1/2 Total Thickness	STD	p value	Bonf α	Bonf sig	Dunn-Sidàk α	sig	Mean Posterior 1/4 Total Thickness	STD	p value	Bonf α	Bonf sig	Dunn-Sidàk α	sig
Mean Total Thickness Tethered	623.787	116.680	0.531	0.003846	no	0.003937864	no	651.572	118.639	0.901	0.003846	no	0.003937864	no	634.068	104.456	0.788	0.003846	no	0.003937864	no
Mean Total Thickness All Untethered	732.885	130.612	0.393	0.003846	no	0.003937864	no	744.338	140.906	0.248	0.003846	no	0.003937864	no	761.226	130.484	0.612	0.003846	no	0.003937864	no
Mean Thickness D and P1	699.517	116.968	0.323	0.003846	no	0.003937864	no	688.696	64.679	0.434	0.003846	no	0.003937864	no	727.623	99.820	0.899	0.003846	no	0.003937864	no
Mean Thickness P2 & P1	775.764	142.518	0.728	0.003846	no	0.003937864	no	783.924	159.545	0.321	0.003846	no	0.003937864	no	774.944	144.869	0.426	0.003846	no	0.003937864	no
Between Tethered and Untethered			0.045	0.003846	no	0.003937864	no			0.098	0.003846	no	0.003937864	no			0.015	0.003846	no	0.003937864	no
Mean Total Thickness Dd	647.126	30.202	0.810	0.003846	no	0.003937864	no	665.167	40.972	0.775	0.003846	no	0.003937864	no	733.792	118.081	0.466	0.003846	no	0.003937864	no
Mean Total Thickness D1	675.894	176.494	0.878	0.003846	no	0.003937864	no	690.691	175.041	0.953	0.003846	no	0.003937864	no	677.258	142.246	0.950	0.003846	no	0.003937864	no
Mean Total Thickness D2	525.536	68.393	0.046	0.003846	no	0.003937864	no	607.332	147.818	0.279	0.003846	no	0.003937864	no	580.259	117.826	0.147	0.003846	no	0.003937864	no
Mean Total Thickness D3	606.227	146.397	0.420	0.003846	no	0.003937864	no	619.498	161.984	0.362	0.003846	no	0.003937864	no	597.852	134.981	0.235	0.003846	no	0.003937864	no
Mean Total Thickness D4	630.464	78.384	0.639	0.003846	no	0.003937864	no	649.701	90.243	0.620	0.003846	no	0.003937864	no	668.053	110.488	0.849	0.003846	no	0.003937864	no
Mean Total Thickness D5	680.813	89.086	0.825	0.003846	no	0.003937864	no	690.640	50.853	0.954	0.003846	no	0.003937864	no	646.919	27.272	0.627	0.003846	no	0.003937864	no
Mean Total Thickness P1	751.908	158.294	0.225	0.003846	no	0.003937864	no	712.225	84.374	0.726	0.003846	no	0.003937864	no	721.453	104.176	0.579	0.003846	no	0.003937864	no
Mean Total Thickness P2	799.620	154.965	0.054	0.003846	no	0.003937864	no	855.624	202.719	0.014	0.003846	no	0.003937864	no	828.434	181.745	0.031	0.003846	no	0.003937864	no

File: EXP 23101 Bioquant Data
Found in 'Proliferative Zone Analysis' tab

Repeat with Proliferative Zone Values — Area marked in GREEN. See image to the right.

	Mean Total Thickness	STD Total Thickness	p value	Bonf α	Bonf sig	Dunn-Sidàk α	sig	Mean Ant Unossified total Thickness	STD	p-value	Bonf α	Bonf sig	Dunn-Sidàk α	sig
Mean Proliferative Thickness Tethered	385.4033	43.3797	0.5141	0.003846	no	0.003937864	no	366.732	44.263	0.395	0.003846	no	0.003937864	no
Mean Proliferative Thickness All Untethered	449.7697	63.3278	0.1479	0.003846	no	0.003937864	no	448.774	109.715	0.803	0.003846	no	0.003937864	no
Mean Proliferative Thickness D and P1	449.7697	63.3278	0.1386	0.003846	no	0.003937864	no	436.930	124.102	0.635	0.003846	no	0.003937864	no
Mean Proliferative Thickness P2 & P1	488.9846	70.5334	0.9873	0.003846	no	0.003937864	no	465.905	122.598	0.965	0.003846	no	0.003937864	no
Between Tethered and Untethered			0.0001	0.003846	yes	0.003937864	yes			0.028	0.003846	no	0.003937864	no
Mean Proliferative Thickness Dd	410.5547	20.9195	0.8918	0.003846	no	0.003937864	no	397.380	45.460	0.972	0.003846	no	0.003937864	no
Mean Total Proliferative Thickness D1	385.4033	43.3797	0.5571	0.003846	no	0.003937864	no	367.340	44.263	0.563	0.003846	no	0.003937864	no
Mean Total Proliferative Thickness D2	352.2358	20.6563	0.1141	0.003846	no	0.003937864	no	382.168	50.596	0.787	0.003846	no	0.003937864	no
Mean Total Proliferative Thickness D3	356.9767	46.3106	0.1519	0.003846	no	0.003937864	no	307.203	55.144	0.058	0.003846	no	0.003937864	no
Mean Total Proliferative Thickness D4	366.0488	21.6830	0.2472	0.003846	no	0.003937864	no	378.803	51.406	0.734	0.003846	no	0.003937864	no
Mean Total Proliferative Thickness D5	398.0738	39.7625	0.8245	0.003846	no	0.003937864	no	398.143	77.840	0.953	0.003846	no	0.003937864	no
Mean Total Proliferative Thickness P1	488.9846	70.5334	0.0102	0.003846	no	0.003937864	no	463.297	164.811	0.151	0.003846	no	0.003937864	no
Mean Total Proliferative Thickness P2	488.2054	37.7223	0.0111	0.003846	no	0.003937864	no	468.513	101.944	0.120	0.003846	no	0.003937864	no

	Mean Ant 1/4 Total Thickness	STD	p-value	Bonf α	Bonf sig	Dunn-Sidàk α	sig	Mean middle 1/2 Total Thickness	STD	p value	Bonf α	Bonf sig	Dunn-Sidàk α	sig	Mean Posterior 1/4 Total Thickness	STD	p value	Bonf α	Bonf sig	Dunn-Sidàk α	sig
Mean Proliferative Thickness Tethered	372.645	35.946	0.742	0.003846	no	0.003937864	no	403.265	61.055	0.496	0.003846	no	0.003937864	no	343.678	57.741	0.506841	0.003846	no	0.003937864	no
Mean Proliferative Thickness All Untethered	364.898	26.528	0.443	0.003846	no	0.003937864	no	495.895	42.122	0.635	0.003846	no	0.003937864	no	531.231	132.068	0.056	0.003846	no	0.003937864	no
Mean Proliferative Thickness D and P1	356.499	20.546	0.733	0.003846	no	0.003937864	no	495.394	51.980	0.453	0.003846	no	0.003937864	no	507.001	160.131	0.075	0.003846	no	0.003937864	no
Mean Proliferative Thickness P2 & P1	370.785	27.561	0.390	0.003846	no	0.003937864	no	505.248	25.320	0.482	0.003846	no	0.003937864	no	599.430	98.606	0.676	0.003846	no	0.003937864	no
Between Tethered and Untethered			0.581	0.003846	no	0.003937864	no			0.001	0.003846	yes	0.003937864	yes			0.0001	0.003846	yes	0.003937864	yes
Mean Proliferative Thickness Dd	353.125	24.577	0.353	0.003846	no	0.003937864	no	477.190	68.607	0.315	0.003846	no	0.003937864	no	394.832	60.000	0.790	0.003846	no	0.003937864	no
Mean Total Proliferative Thickness D1	379.762	48.652	0.578	0.003846	no	0.003937864	no	417.943	69.309	0.610	0.003846	no	0.003937864	no	376.568	62.030	0.603	0.003846	no	0.003937864	no
Mean Total Proliferative Thickness D2	391.773	41.896	0.214	0.003846	no	0.003937864	no	349.757	42.955	0.017	0.003846	no	0.003937864	no	307.520	32.094	0.129728	0.003846	no	0.003937864	no
Mean Total Proliferative Thickness D3	359.545	24.705	0.571	0.003846	no	0.003937864	no	416.083	86.265	0.577	0.003846	no	0.003937864	no	319.440	73.039	0.181	0.003846	no	0.003937864	no
Mean Total Proliferative Thickness D4	353.452	40.983	0.363	0.003846	no	0.003937864	no	393.403	64.310	0.251	0.003846	no	0.003937864	no	338.537	34.318	0.289	0.003846	no	0.003937864	no
Mean Total Proliferative Thickness D5	378.692	33.805	0.619	0.003846	no	0.003937864	no	439.137	30.452	0.977	0.003846	no	0.003937864	no	376.323	76.452	0.600	0.003846	no	0.003937864	no
Mean Total Proliferative Thickness P1	359.873	20.425	0.584	0.003846	no	0.003937864	no	513.598	32.459	0.045	0.003846	no	0.003937864	no	619.170	150.860	0.001	0.003846	yes	0.003937864	yes
Mean Total Proliferative Thickness P2	381.697	33.536	0.506	0.003846	no	0.003937864	no	496.897	18.437	0.125	0.003846	no	0.003937864	no	579.690	19.489	0.014	0.003846	no	0.003937864	no

File: EXP 23101 Bioquant Data
Found in 'Hypertrophic Zone Analysis' tab

Repeat with Hypertrophic Zone Values — Area marked in RED. See image to the right.

	Mean Total Thickness	STD Total Thickness	p value	Bonf α	Bonf sig	Dunn-Sidàk α	sig	Mean Ant Unossified total Thickness	STD	p-value	Bonf α	Bonf sig	Dunn-Sidàk α	sig
Mean Hypertrophic Thickness Tethered	252.218	16.676	0.425	0.003846	no	0.003937864	no	266.790	34.135	0.878	0.003846	no	0.003937864	no
Mean Hypertrophic Thickness All Untethered	252.799	73.072	0.446	0.003846	no	0.003937864	no	237.167	74.338	0.570	0.003846	no	0.003937864	no
Mean Hypertrophic Thickness D and P1	230.488	40.190	0.467	0.003846	no	0.003937864	no	214.434	48.804	0.613	0.003846	no	0.003937864	no
Mean Hypertrophic Thickness P2 & P1	257.114	87.103	0.305	0.003846	no	0.003937864	no	239.241	84.025	0.351	0.003846	no	0.003937864	no
Between Tethered and Untethered			0.976	0.003846	no	0.003937864	no			0.200	0.003846	no	0.003937864	no
Mean Total Hypertrophic Thickness Dd	244.170	47.147	0.742	0.003846	no	0.003937864	no	230.945	57.254	0.480	0.003846	no	0.003937864	no
Mean Total Hypertrophic Thickness D1	255.644	7.927	0.898	0.003846	no	0.003937864	no	253.457	48.744	0.917	0.003846	no	0.003937864	no
Mean Total Hypertrophic Thickness D2	266.606	24.380	0.572	0.003846	no	0.003937864	no	266.403	11.733	0.732	0.003846	no	0.003937864	no
Mean Total Hypertrophic Thickness D3	247.991	25.787	0.860	0.003846	no	0.003937864	no	257.132	31.293	0.982	0.003846	no	0.003937864	no
Mean Total Hypertrophic Thickness D4	240.050	3.769	0.621	0.003846	no	0.003937864	no	275.185	48.776	0.517	0.003846	no	0.003937864	no
Mean Total Hypertrophic Thickness D5	250.798	5.491	0.948	0.003846	no	0.003937864	no	281.775	37.883	0.379	0.003846	no	0.003937864	no
Mean Total Hypertrophic Thickness P1	216.806	35.409	0.146	0.003846	no	0.003937864	no	203.427	51.675	0.056	0.003846	no	0.003937864	no
Mean Total Hypertrophic Thickness P2	297.421	113.312	0.063	0.003846	no	0.003937864	no	275.055	105.510	0.520	0.003846	no	0.003937864	no

	Mean Ant 1/4 Total Thickness	STD	p-value	Bonf α	Bonf sig	Dunn-Sidàk α	sig	Mean middle 1/2 Total Thickness	STD	p value	Bonf α	Bonf sig	Dunn-Sidàk α	sig	Mean Posterior 1/4 Total Thickness	STD	p value	Bonf α	Bonf sig	Dunn-Sidàk α	sig
Mean Hypertrophic Thickness Tethered	242.739	27.984	0.828	0.003846	no	0.003937864	no	248.948	20.461	0.156	0.003846	no	0.003937864	no	243.120	30.388	0.109	0.003846	no	0.003937864	no
Mean Hypertrophic Thickness All Untethered	221.750	80.230	0.643	0.003846	no	0.003937864	no	269.255	81.189	0.357	0.003846	no	0.003937864	no	265.530	80.729	0.645	0.003846	no	0.003937864	no
Mean Hypertrophic Thickness D and P1	202.251	61.159	0.840	0.003846	no	0.003937864	no	241.263	38.159	0.417	0.003846	no	0.003937864	no	245.905	54.509	0.842	0.003846	no	0.003937864	no
Mean Hypertrophic Thickness P2 & P1	228.503	82.337	0.396	0.003846	no	0.003937864	no	276.074	99.200	0.266	0.003846	no	0.003937864	no	272.706	100.220	0.496	0.003846	no	0.003937864	no
Between Tethered and Untethered			0.360	0.003846	no	0.003937864	no			0.361	0.003846	no	0.003937864	no			0.339	0.003846	no	0.003937864	no
Mean Total Hypertrophic Thickness Dd	208.243	91.590	0.365	0.003846	no	0.003937864	no	255.617	36.695	0.974	0.003846	no	0.003937864	no	251.178	22.246	0.991	0.003846	no	0.003937864	no
Mean Total Hypertrophic Thickness D1	243.762	26.905	0.764	0.003846	no	0.003937864	no	252.450	6.031	0.886	0.003846	no	0.003937864	no	272.907	23.080	0.478	0.003846	no	0.003937864	no
Mean Total Hypertrophic Thickness D2	258.943	16.357	0.414	0.003846	no	0.003937864	no	263.898	29.800	0.798	0.003846	no	0.003937864	no	251.327	23.188	0.995	0.003846	no	0.003937864	no
Mean Total Hypertrophic Thickness D3	243.042	53.112	0.783	0.003846	no	0.003937864	no	261.395	4.353	0.866	0.003846	no	0.003937864	no	219.877	32.345	0.291	0.003846	no	0.003937864	no
Mean Total Hypertrophic Thickness D4	229.023	25.173	0.844	0.003846	no	0.003937864	no	236.850	18.762	0.490	0.003846	no	0.003937864	no	219.142	18.006	0.279	0.003846	no	0.003937864	no
Mean Total Hypertrophic Thickness D5	238.923	18.338	0.891	0.003846	no	0.003937864	no	230.145	17.295	0.353	0.003846	no	0.003937864	no	252.348	28.121	0.978	0.003846	no	0.003937864	no
Mean Total Hypertrophic Thickness P1	196.258	29.233	0.184	0.003846	no	0.003937864	no	226.908	40.934	0.296	0.003846	no	0.003937864	no	240.632	82.764	0.719	0.003846	no	0.003937864	no
Mean Total Hypertrophic Thickness P2	260.748	113.906	0.379	0.003846	no	0.003937864	no	325.240	125.196	0.010	0.003846	no	0.003937864	no	304.780	123.183	0.068	0.003846	no	0.003937864	no

File: Stats.GR.Prox vs Distal Physis.Longtether
Found in 'Low Tension %GM' tab

% Growth Modulation	Mean % Growth Modulation	STD Total Thickness	p value	Bonf α	Bonf sig	Dunn-Sidàk α	sig
Mean %GM Tethered	25%	24%	3.2E-03	0.003846	yes	0.003937864	yes
Mean %GM Untethered	-41%	23%	0.159	0.003846	no	0.003937864	no
Mean %GM D and P1	-48%	24%	0.540	0.003846	no	0.003937864	no
Mean %GM P2 & P1	-40%	26%	0.099	0.003846	no	0.003937864	no
Between Tethered and Untethered			1.83383E-12	0.003846	yes	0.003937864	yes
Mean %GM Dd	-43%	16%	0.0030	0.003846	yes	0.003937864	yes
Mean %GM D1	14%	16%	0.3928	0.003846	no	0.003937864	no
Mean %GM D2	10%	19%	0.5293	0.003846	no	0.003937864	no
Mean %GM D3	22%	16%	0.1636	0.003846	no	0.003937864	no
Mean %GM D4	27%	18%	0.0863	0.003846	no	0.003937864	no
Mean %GM D5	55%	24%	0.0002	0.003846	yes	0.003937864	yes
Mean %GM P1	-53%	31%	0.0002	0.003846	yes	0.003937864	yes
Mean %GM P2	-27%	12%	0.0672	0.003846	no	0.003937864	no

File: Stats.GR.Prox vs Distal Physis.Longtether
Found in 'Low Tension %GM' tab

Mean Vertebral Growth Rates	Mean Growth Rates	STD Total Thickness	p value	Bonf α	Bonf sig	Dunn-Sidàk α	sig
Mean Vertebral Growth Rate Tethered	81.335	13.60109	0.197	0.003846	no	0.003937864	no
Mean Vertebral Growth Rate Untethered	78.3545	12.09306	0.476	0.003846	no	0.003937864	no
Mean Vertebral Growth Rate D and P1	78.75988	12.12239	0.229	0.003846	no	0.003937864	no
Mean Vertebral Growth Rate P2 & P1	75.97191	13.23076	0.701	0.003846	no	0.003937864	no
Between Tethered and Untethered			0.448	0.003846	no	0.003937864	no
Mean Vertebral Growth Rate Dd	83.11969	8.440042	0.565	0.003846	no	0.003937864	no
Mean Vertebral Growth Rate D1	88.5989	14.77297	0.092	0.003846	no	0.003937864	no
Mean Vertebral Growth Rate D2	86.76621	14.17906	0.190	0.003846	no	0.003937864	no
Mean Vertebral Growth Rate D3	82.14399	10.34709	0.702	0.003846	no	0.003937864	no
Mean Vertebral Growth Rate D4	76.96249	13.35238	0.518	0.003846	no	0.003937864	no
Mean Vertebral Growth Rate D5	72.20343	12.0748	0.107	0.003846	no	0.003937864	no
Mean Vertebral Growth Rate P1	74.40008	14.3681	0.246	0.003846	no	0.003937864	no
Mean Vertebral Growth Rate P2	77.54373	13.14325	0.596	0.003846	no	0.003937864	no

File: Stats.GR.Prox vs Distal Physis.Longtether
Found in 'Low Tension %GM' tab

Prox vs Distal Mean Growth Rates	Distal Physis							Proximal Physis						
	Mean Growth Rate	STD Total Thickness	p value	Bonf α	Bonf sig	Dunn-Sidàk α	sig	Mean Growth Rate	STD Total Thickness	p value	Bonf α	Bonf sig	Dunn-Sidàk α	sig
Mean Growth Rate Tethered	83.23442	15.0085	0.598	0.003846	no	0.003937864	no	79.43558	12.2559	0.618	0.003846	no	0.003937864	no
Mean Growth Rate Untethered	84.98017	7.971383	0.798	0.003846	no	0.003937864	no	71.72883	12.18392	0.236	0.003846	no	0.003937864	no
Mean Growth Rate D and P1	83.64044	8.70488	0.838	0.003846	no	0.003937864	no	73.87933	13.79684	0.185	0.003846	no	0.003937864	no
Mean Growth Rate P2 & P1	85.22031	9.296092	0.581	0.003846	no	0.003937864	no	66.7235	9.66541	0.881	0.003846	no	0.003937864	no
Between Tethered and Untethered			0.751	0.003846	no	0.003937864	no			0.149	0.003846	no	0.003937864	no
Mean Growth Rate Dd	84.49989	6.13266	0.734	0.003846	no	0.003937864	no	81.73949	11.60868					
Mean Growth Rate D1	92.10991	17.97903	0.618	0.003846	no	0.003937864	no	85.08789	13.61522					
Mean Growth Rate D2	89.69646	18.84009	0.666	0.003846	no	0.003937864	no	83.83596	11.04111					
Mean Growth Rate D3	82.15111	10.49746	0.999	0.003846	no	0.003937864	no	82.13687	12.54825					
Mean Growth Rate D4	78.51849	15.34511	0.810	0.003846	no	0.003937864	no	75.40648	14.24708					
Mean Growth Rate D5	73.69615	14.16101	0.798	0.003846	no	0.003937864	no	70.71071	12.54124					
Mean Growth Rate P1	82.78099	12.23159	0.172	0.003846	no	0.003937864	no	66.01917	12.48098					
Mean Growth Rate P2	87.65963	6.969974	0.035	0.003846	no	0.003937864	no	67.42783	8.734265					

File: Stats.GR.Prox vs Distal Physis.Longtether
Found in 'Low Tension %GM' tab

Prox vs Distal % Growth Modulation	Distal Physis							Proximal Physis						
	Mean % Growth Modulation	STD Total Thickness	p value	Bonf α	Bonf sig	Dunn-Sidàk α	sig	Mean % Growth Modulation	STD Total Thickness	p value	Bonf α	Bonf sig	Dunn-Sidàk α	sig
Mean %GM Tethered	24%	23%	0.139	0.003846	no	0.003937864	no	27%	25%	0.063	0.003846	no	0.003937864	no
Mean %GM Untethered	-51%	25%	0.013	0.003846	no	0.003937864	no	-32%	17%	0.315	0.003846	no	0.003937864	no
Mean %GM D and P1	-61%	22%	0.015	0.003846	no	0.003937864	no	-35%	20%	0.314	0.003846	no	0.003937864	no
Mean %GM P2 & P1	-55%	30%	0.018	0.003846	no	0.003937864	no	-25%	10%	0.879	0.003846	no	0.003937864	no
Between Tethered and Untethered			1.98E-07	0.003846	yes	0.003937864	yes			2.97E-06	0.003846	yes	0.003937864	yes
Mean %GM Dd	-43%	11%	0.931	0.003846	no	0.003937864	no	-44%	23%					
Mean %GM D1	12%	14%	0.865	0.003846	no	0.003937864	no	15%	21%					
Mean %GM D2	6%	24%	0.687	0.003846	no	0.003937864	no	14%	18%					
Mean %GM D3	30%	15%	0.216	0.003846	no	0.003937864	no	13%	14%					
Mean %GM D4	19%	12%	0.359	0.003846	no	0.003937864	no	34%	23%					
Mean %GM D5	49%	29%	0.639	0.003846	no	0.003937864	no	60%	22%					
Mean %GM P1	-79%	11%	0.008	0.003846	no	0.003937864	no	-26%	15%					
Mean %GM P2	-30%	19%	0.628	0.003846	no	0.003937864	no	-25%	4%					

High Tension	1206-2	1212-1	1219-1			

Nucleus Pulposis Location

alpha 0.05; k 6 Reported p-values (both tables)

	%=distance Posterior VB to midpoint NP							% distance Posterior **ossified** epiphysis to midpoint NP						
	Mean	STD	P-value	Bonf α	Bonf sig	Dunn-Sidàk α	sig	Mean	STD	P-value	Bonf α	Bonf sig	Dunn-Sidàk α	sig
Mean % Across All Tethered	42.20%	5.72%	0.950	0.008333	no	0.008512445	no	45.31%	5.6%	0.718	0.008333	no	0.008512445	no
Mean % Across all Untethered	34.11%	4.34%	0.037	0.008333	no	0.008512445	no	43.12%	3.6%	0.145	0.008333	no	0.008512445	no
Mean %Across D and P1	31.79%	2.49%	0.516	0.008333	no	0.008512445	no	41.63%	2.5%	0.255	0.008333	no	0.008512445	no
Mean % Across P2	34.88%	4.75%	0.018	0.008333	no	0.008512445	no	43.24%	4.2%	0.095	0.008333	no	0.008512445	no
Between Tethered and Untethered			0.004	0.008333	yes	0.008512445	yes			0.335	0.008333	no	0.008512445	no
Mean % across Dd	32.55%	3.66%	0.100	0.008333	no	0.008512445	no	42.87%	2.6%	0.602	0.008333	no	0.008512445	no
Mean % Across D1	41.62%	6.79%	0.323	0.008333	no	0.008512445	no	43.56%	6.3%	0.798	0.008333	no	0.008512445	no
Mean % Across D2 (NA)														
Mean % Across D3	43.19%	8.60%	0.143	0.008333	no	0.008512445	no	44.78%	8.1%	0.828	0.008333	no	0.008512445	no
Mean % Across D4	41.79%	2.95%	0.298	0.008333	no	0.008512445	no	47.61%	2.4%	0.176	0.008333	no	0.008512445	no
Mean % Across D5 (NA)														
Mean % Across P1	31.03%	0.66%	0.031	0.008333	no	0.008512445	no	40.38%	2.0%	0.124	0.008333	no	0.008512445	no
Mean % Across P2	38.74%	3.37%	0.869	0.008333	no	0.008512445	no	46.10%	4.1%	0.463	0.008333	no	0.008512445	no

Epiphyseal Percent Ossification

Define

alpha 0.05; k 8 Reported p-values

**% Epiphyseal Ossification**	Mean	STD	P-value	Bonf α	Bonf sig	Dunn-Sidàk α	sig
Mean % Across All Tethered	93.21%	6.19%	0.211	0.00625	no	0.006391151	no
Mean % Across all Untethered	78.95%	5.31%	0.094	0.00625	no	0.006391151	no
Mean %Across D and P1	76.36%	3.54%	0.748	0.00625	no	0.006391151	no
Mean % Across P2 & P1	80.52%	5.09%	0.068	0.00625	no	0.006391151	no
Between Tethered and Untethered			0.000	0.00625	yes	0.006391151	yes
Mean % across Dd and D1	75.81%	5.08%	0.030	0.00625	no	0.006391151	no
Mean % Across D1	95.41%	3.62%	0.052	0.00625	no	0.006391151	no
Mean % Across D2 (NA)							
Mean % Across D3	96.24%	1.87%	0.032	0.00625	no	0.006391151	no
Mean % Across D4	87.99%	8.64%	0.708	0.00625	no	0.006391151	no
Mean % Across D5 (NA)							
Mean % Across P1	76.91%	2.14%	0.056	0.00625	no	0.006391151	no
Mean % Across P2	84.14%	4.57%	0.702	0.00625	no	0.006391151	no

Growth Plate Analysis

File: EXP 23101 Bioquant Data
Found in 'GP Analysis' tab
alpha 0.05; k 13 Reported p-values

	Mean Total Thickness	STD Total Thickness	p value	Bonf α	Bonf sig	Dunn-Sidàk α	sig	Mean Ant Unossified total Thickness	STD	p-value	Bonf α	Bonf sig	Dunn-Sidàk α	sig
Mean Total Thickness Tethered	615.645	95.368	0.802	0.003846	no	0.003937864	no	522.546	103.781	0.344	0.003846	no	0.003937864	no
Mean Total Thickness All Untethered	724.203	99.222	0.591	0.003846	no	0.003937864	no	655.228	77.607	0.150	0.003846	no	0.003937864	no
Mean Thickness D and P1	718.142	109.728	0.375	0.003846	no	0.003937864	no	625.557	70.917	0.162	0.003846	no	0.003937864	no
Mean Thickness P2 & P1	749.600	88.600	0.756	0.003846	no	0.003937864	no	684.003	52.527	0.393	0.003846	no	0.003937864	no
Between Tethered and Untethered			0.014	0.003846	no	0.003937864	no			0.005	0.003846	no	0.003937864	no
Mean Total Thickness Dd	673.409	118.110	0.779	0.003846	no	0.003937864	no	568.904	92.155	0.998	0.003846	no	0.003937864	no
Mean Total Thickness D1	648.854	99.612	0.902	0.003846	no	0.003937864	no	615.562	171.427	0.457	0.003846	no	0.003937864	no
Mean Total Thickness D2	569.512	129.978	0.143	0.003846	no	0.003937864	no	441.119	23.016	0.034	0.003846	no	0.003937864	no
Mean Total Thickness D3	580.575	62.236	0.204	0.003846	no	0.003937864	no	531.824	33.866	0.560	0.003846	no	0.003937864	no
Mean Total Thickness D4	651.518	85.351	0.937	0.003846	no	0.003937864	no	486.011	43.975	0.183	0.003846	no	0.003937864	no
Mean Total Thickness D5	627.768	129.801	0.637	0.003846	no	0.003937864	no	538.216	130.281	0.630	0.003846	no	0.003937864	no
Mean Total Thickness P1	762.875	100.735	0.069	0.003846	no	0.003937864	no	663.326	21.514	0.125	0.003846	no	0.003937864	no
Mean Total Thickness P2	736.324	94.598	0.179	0.003846	no	0.003937864	no	704.680	71.778	0.023	0.003846	no	0.003937864	no

	Mean Ant 1/4 Total Thickness	STD	p-value	Bonf α	Bonf sig	Dunn-Sidàk α	sig	Mean middle 1/2 Total Thickness	STD	p value	Bonf α	Bonf sig	Dunn-Sidàk α	sig
Mean Total Thickness Tethered	609.904	99.085	0.939	0.003846	no	0.003937864	no	679.069	131.671	0.948	0.003846	no	0.003937864	no
Mean Total Thickness All Untethered	682.570	102.496	0.775	0.003846	no	0.003937864	no	775.388	141.427	0.899	0.003846	no	0.003937864	no
Mean Thickness D and P1	693.096	126.695	0.638	0.003846	no	0.003937864	no	789.248	163.635	0.812	0.003846	no	0.003937864	no
Mean Thickness P2 & P1	691.540	117.695	0.592	0.003846	no	0.003937864	no	777.875	116.490	0.585	0.003846	no	0.003937864	no
Between Tethered and Untethered			0.100	0.003846	no	0.003937864	no			0.105	0.003846	no	0.003937864	no
Mean Total Thickness Dd	664.629	81.655	0.637	0.003846	no	0.003937864	no	770.413	214.536	0.479	0.003846	no	0.003937864	no
Mean Total Thickness D1	598.928	52.612	0.510	0.003846	no	0.003937864	no	702.030	151.569	0.867	0.003846	no	0.003937864	no
Mean Total Thickness D2	589.564	147.637	0.411	0.003846	no	0.003937864	no	630.687	177.312	0.275	0.003846	no	0.003937864	no
Mean Total Thickness D3	582.754	65.594	0.346	0.003846	no	0.003937864	no	656.110	156.098	0.449	0.003846	no	0.003937864	no
Mean Total Thickness D4	628.674	115.233	0.884	0.003846	no	0.003937864	no	721.089	139.201	0.940	0.003846	no	0.003937864	no
Mean Total Thickness D5	649.598	147.580	0.831	0.003846	no	0.003937864	no	685.426	124.104	0.704	0.003846	no	0.003937864	no
Mean Total Thickness P1	721.562	176.154	0.138	0.003846	no	0.003937864	no	808.083	140.892	0.229	0.003846	no	0.003937864	no
Mean Total Thickness P2	661.518	29.931	0.676	0.003846	no	0.003937864	no	747.667	106.473	0.679	0.003846	no	0.003937864	no

	Mean Posterior 1/4 Total Thickness	STD	p value	Bonf α	Bonf sig	Dunn-Sidàk α	sig
Mean Total Thickness Tethered	627.381	115.510	0.778	0.003846	no	0.003937864	no
Mean Total Thickness All Untethered	801.138	213.877	0.534	0.003846	no	0.003937864	no
Mean Thickness D and P1	752.973	197.346	0.465	0.003846	no	0.003937864	no
Mean Thickness P2 & P1	858.914	195.453	0.681	0.003846	no	0.003937864	no
Between Tethered and Untethered			0.016	0.003846	no	0.003937864	no
Mean Total Thickness Dd	685.587	239.638	0.944	0.003846	no	0.003937864	no
Mean Total Thickness D1	650.377	103.040	0.670	0.003846	no	0.003937864	no
Mean Total Thickness D2	616.678	186.345	0.440	0.003846	no	0.003937864	no
Mean Total Thickness D3	551.611	33.437	0.145	0.003846	no	0.003937864	no
Mean Total Thickness D4	680.405	82.577	0.902	0.003846	no	0.003937864	no
Mean Total Thickness D5	637.831	161.744	0.579	0.003846	no	0.003937864	no
Mean Total Thickness P1	820.359	162.218	0.188	0.003846	no	0.003937864	no
Mean Total Thickness P2	897.469	254.422	0.029	0.003846	no	0.003937864	no

Repeat with Proliferative Zone Values

File: EXP 23101 Bioquant Data
Found in 'Proliferative Zone Analysis' tab
Area marked in GREEN. See image to the right.

	Mean Total Thickness	STD Total Thickness	p value	Bonf α	Bonf sig	Dunn-Sidàk α	sig	Mean Ant Unossified total Thickness	STD	p-value	Bonf α	Bonf sig	Dunn-Sidàk α	sig
Mean Proliferative Thickness Tethered	371.147	35.355	0.671	0.003846	no	0.003937864	no	317.485	39.962	0.237	0.003846	no	0.003937864	no
Mean Proliferative Thickness All Untethered	414.949	34.683	0.721	0.003846	no	0.003937864	no	373.477	35.228	0.093	0.003846	no	0.003937864	no
Mean Proliferative Thickness D and P1	422.120	40.544	0.865	0.003846	no	0.003937864	no	354.631	24.480	0.395	0.003846	no	0.003937864	no
Mean Proliferative Thickness P2 & P1	409.688	31.364	0.540	0.003846	no	0.003937864	no	379.461	44.855	0.123	0.003846	no	0.003937864	no
Between Tethered and Untethered			0.007	0.003846	no	0.003937864	no			0.005	0.003846	no	0.003937864	no
Mean Proliferative Thickness Dd	425.470	45.862	0.084	0.003846	no	0.003937864	no	363.503	8.994	0.355	0.003846	no	0.003937864	no
Mean Total Proliferative Thickness D1	374.364	16.959	0.559	0.003846	no	0.003937864	no	346.090	10.324	0.810	0.003846	no	0.003937864	no
Mean Total Proliferative Thickness D2	380.922	32.227	0.769	0.003846	no	0.003937864	no	343.120	14.128	0.921	0.003846	no	0.003937864	no
Mean Total Proliferative Thickness D3	381.606	58.103	0.792	0.003846	no	0.003937864	no	324.095	39.294	0.627	0.003846	no	0.003937864	no
Mean Total Proliferative Thickness D4	341.548	48.466	0.032	0.003846	no	0.003937864	no	275.413	64.400	0.035	0.003846	no	0.003937864	no
Mean Total Proliferative Thickness D5	377.293	2.248	0.650	0.003846	no	0.003937864	no	295.432	36.102	0.072	0.003846	no	0.003937864	no
Mean Total Proliferative Thickness P1	418.770	44.413	0.159	0.003846	no	0.003937864	no	341.323	40.556	0.965	0.003846	no	0.003937864	no
Mean Total Proliferative Thickness P2	400.607	15.471	0.564	0.003846	no	0.003937864	no	404.887	27.882	0.005	0.003846	no	0.003937864	no

	Mean Ant 1/4 Total Thickness	STD	p-value	Bonf α	Bonf sig	Dunn-Sidàk α	sig	Mean middle 1/2 Total Thickness	STD	p value	Bonf α	Bonf sig	Dunn-Sidàk α	sig
Mean Proliferative Thickness Tethered	373.578	41.791	0.816	0.003846	no	0.003937864	no	395.977	49.505	0.147	0.003846	no	0.003937864	no
Mean Proliferative Thickness All Untethered	365.306	44.446	0.684	0.003846	no	0.003937864	no	424.441	45.729	0.632	0.003846	no	0.003937864	no
Mean Proliferative Thickness D and P1	368.573	46.523	0.431	0.003846	no	0.003937864	no	432.818	54.132	0.598	0.003846	no	0.003937864	no
Mean Proliferative Thickness P2 & P1	351.039	47.214	0.851	0.003846	no	0.003937864	no	427.047	35.452	0.210	0.003846	no	0.003937864	no
Between Tethered and Untethered			0.676	0.003846	no	0.003937864	no			0.175	0.003846	no	0.003937864	no
Mean Proliferative Thickness Dd	384.330	40.406	0.563	0.003846	no	0.003937864	no	419.228	71.842	0.646	0.003846	no	0.003937864	no
Mean Total Proliferative Thickness D1	368.725	55.308	0.924	0.003846	no	0.003937864	no	356.318	7.596	0.056	0.003846	no	0.003937864	no
Mean Total Proliferative Thickness D2	388.738	26.909	0.441	0.003846	no	0.003937864	no	385.815	55.535	0.445	0.003846	no	0.003937864	no
Mean Total Proliferative Thickness D3	389.550	72.590	0.420	0.003846	no	0.003937864	no	449.278	64.742	0.110	0.003846	no	0.003937864	no
Mean Total Proliferative Thickness D4	349.710	24.539	0.356	0.003846	no	0.003937864	no	374.427	39.707	0.233	0.003846	no	0.003937864	no
Mean Total Proliferative Thickness D5	371.168	30.740	0.992	0.003846	no	0.003937864	no	414.048	16.820	0.787	0.003846	no	0.003937864	no
Mean Total Proliferative Thickness P1	344.938	59.415	0.368	0.003846	no	0.003937864	no	446.407	40.129	0.137	0.003846	no	0.003937864	no
Mean Total Proliferative Thickness P2	357.140	54.850	0.636	0.003846	no	0.003937864	no	407.687	20.183	0.970	0.003846	no	0.003937864	no

	Mean Posterior 1/4 Total Thickness	STD	p value	Bonf α	Bonf sig	Dunn-Sidàk α	sig
Mean Proliferative Thickness Tethered	368.872	70.466	0.280	0.003846	no	0.003937864	no
Mean Proliferative Thickness All Untethered	486.986	87.334	0.550	0.003846	no	0.003937864	no
Mean Proliferative Thickness D and P1	504.453	99.297	0.516	0.003846	no	0.003937864	no
Mean Proliferative Thickness P2 & P1	463.069	74.091	0.758	0.003846	no	0.003937864	no
Between Tethered and Untethered			0.001	0.003846	yes	0.003937864	yes
Mean Proliferative Thickness Dd	534.820	107.876	0.014	0.003846	no	0.003937864	no
Mean Total Proliferative Thickness D1	426.322	51.075	0.805	0.003846	no	0.003937864	no
Mean Total Proliferative Thickness D2	402.102	116.987	0.835	0.003846	no	0.003937864	no
Mean Total Proliferative Thickness D3	322.032	51.400	0.076	0.003846	no	0.003937864	no
Mean Total Proliferative Thickness D4	323.975	51.580	0.083	0.003846	no	0.003937864	no
Mean Total Proliferative Thickness D5	369.932	15.432	0.413	0.003846	no	0.003937864	no
Mean Total Proliferative Thickness P1	474.087	101.224	0.245	0.003846	no	0.003937864	no
Mean Total Proliferative Thickness P2	452.052	55.796	0.462	0.003846	no	0.003937864	no

Repeat with Hypertrophic Zone Values

File: EXP 23101 Bioquant Data
Found in 'Hypertrophic Zone Analysis' tab
Area marked in RED. See image to the right.

	Mean Total Thickness	STD Total Thickness	p value	Bonf α	Bonf sig	Dunn-Sidàk α	sig	Mean Ant Unossified total Thickness	STD	p-value	Bonf α	Bonf sig	Dunn-Sidàk α	sig
Mean Hypertrophic Thickness Tethered	281.896	27.839	0.840	0.003846	no	0.003937864	no	289.332	53.531	0.857	0.003846	no	0.003937864	no
Mean Hypertrophic Thickness All Untethered	272.606	23.540	0.525	0.003846	no	0.003937864	no	231.007	45.750	0.574	0.003846	no	0.003937864	no
Mean Hypertrophic Thickness D and P1	268.335	26.226	0.443	0.003846	no	0.003937864	no	217.001	44.763	0.756	0.003846	no	0.003937864	no
Mean Hypertrophic Thickness P2 & P1	279.430	26.117	0.892	0.003846	no	0.003937864	no	235.625	40.777	0.256	0.003846	no	0.003937864	no
Between Tethered and Untethered			0.412	0.003846	no	0.003937864	no			0.021	0.003846	no	0.003937864	no
Mean Total Hypertrophic Thickness Dd	258.958	9.598	0.174	0.003846	no	0.003937864	no	223.310	62.113	0.168	0.003846	no	0.003937864	no
Mean Total Hypertrophic Thickness D1	289.667	19.420	0.438	0.003846	no	0.003937864	no	259.755	59.984	0.844	0.003846	no	0.003937864	no
Mean Total Hypertrophic Thickness D2	285.564	47.093	0.624	0.003846	no	0.003937864	no	304.530	77.117	0.330	0.003846	no	0.003937864	no
Mean Total Hypertrophic Thickness D3	290.181	34.339	0.417	0.003846	no	0.003937864	no	317.428	12.449	0.189	0.003846	no	0.003937864	no
Mean Total Hypertrophic Thickness D4	279.279	16.952	0.953	0.003846	no	0.003937864	no	282.070	18.965	0.688	0.003846	no	0.003937864	no
Mean Total Hypertrophic Thickness D5	264.790	26.472	0.347	0.003846	no	0.003937864	no	294.888	80.624	0.358	0.003846	no	0.003937864	no
Mean Total Hypertrophic Thickness P1	277.712	36.928	0.962	0.003846	no	0.003937864	no	207.538	0.456	0.132	0.003846	no	0.003937864	no
Mean Total Hypertrophic Thickness P2	281.149	18.241	0.852	0.003846	no	0.003937864	no	254.351	44.839	0.714	0.003846	no	0.003937864	no

	Mean Ant 1/4 Total Thickness	STD	p-value	Bonf α	Bonf sig	Dunn-Sidàk α	sig	Mean middle 1/2 Total Thickness	STD	p value	Bonf α	Bonf sig	Dunn-Sidàk α	sig
Mean Hypertrophic Thickness Tethered	290.275	47.853	0.411	0.003846	no	0.003937864	no	270.561	32.319	0.896	0.003846	no	0.003937864	no
Mean Hypertrophic Thickness All Untethered	272.845	55.014	0.113	0.003846	no	0.003937864	no	277.372	43.333	0.241	0.003846	no	0.003937864	no
Mean Hypertrophic Thickness D and P1	246.913	30.391	0.656	0.003846	no	0.003937864	no	293.321	45.150	0.525	0.003846	no	0.003937864	no
Mean Hypertrophic Thickness P2 & P1	287.739	65.273	0.117	0.003846	no	0.003937864	no	276.165	54.759	0.195	0.003846	no	0.003937864	no
Between Tethered and Untethered			0.456	0.003846	no	0.003937864	no			0.664	0.003846	no	0.003937864	no
Mean Total Hypertrophic Thickness Dd	252.987	40.665	0.242	0.003846	no	0.003937864	no	279.785	1.155	0.740	0.003846	no	0.003937864	no
Mean Total Hypertrophic Thickness D1	269.292	62.699	0.575	0.003846	no	0.003937864	no	286.605	17.844	0.501	0.003846	no	0.003937864	no
Mean Total Hypertrophic Thickness D2	295.097	43.208	0.707	0.003846	no	0.003937864	no	273.303	49.659	0.993	0.003846	no	0.003937864	no
Mean Total Hypertrophic Thickness D3	312.617	60.708	0.306	0.003846	no	0.003937864	no	271.007	43.144	0.917	0.003846	no	0.003937864	no
Mean Total Hypertrophic Thickness D4	320.857	29.403	0.181	0.003846	no	0.003937864	no	263.328	32.180	0.626	0.003846	no	0.003937864	no
Mean Total Hypertrophic Thickness D5	253.513	28.409	0.250	0.003846	no	0.003937864	no	258.563	30.632	0.467	0.003846	no	0.003937864	no
Mean Total Hypertrophic Thickness P1	237.803	10.504	0.166	0.003846	no	0.003937864	no	306.857	67.419	0.083	0.003846	no	0.003937864	no
Mean Total Hypertrophic Thickness P2	337.675	51.930	0.115	0.003846	no	0.003937864	no	245.473	11.194	0.160	0.003846	no	0.003937864	no

	Mean Posterior 1/4 Total Thickness	STD	p value	Bonf α	Bonf sig	Dunn-Sidàk α	sig
Mean Hypertrophic Thickness Tethered	287.068	48.183	0.245	0.003846	no	0.003937864	no
Mean Hypertrophic Thickness All Untethered	300.252	39.769	0.440	0.003846	no	0.003937864	no
Mean Hypertrophic Thickness D and P1	288.245	31.611	0.571	0.003846	no	0.003937864	no
Mean Hypertrophic Thickness P2 & P1	310.504	45.163	0.518	0.003846	no	0.003937864	no
Between Tethered and Untethered			0.497	0.003846	no	0.003937864	no
Mean Total Hypertrophic Thickness Dd	279.748	16.768	0.623	0.003846	no	0.003937864	no
Mean Total Hypertrophic Thickness D1	343.017	79.003	0.032	0.003846	no	0.003937864	no
Mean Total Hypertrophic Thickness D2	287.162	41.331	0.846	0.003846	no	0.003937864	no
Mean Total Hypertrophic Thickness D3	275.325	28.893	0.502	0.003846	no	0.003937864	no
Mean Total Hypertrophic Thickness D4	264.163	16.015	0.258	0.003846	no	0.003937864	no
Mean Total Hypertrophic Thickness D5	265.672	28.884	0.286	0.003846	no	0.003937864	no
Mean Total Hypertrophic Thickness P1	296.742	44.726	0.850	0.003846	no	0.003937864	no
Mean Total Hypertrophic Thickness P2	324.267	50.306	0.188	0.003846	no	0.003937864	no

% Growth Modulation

	Mean % Growth Modulation	STD Total Thickness	p value	Bonf α	Bonf sig	Dunn-Sidàk α	sig
Mean %GM Tethered	21%	30%	0.011	0.003846	no	0.003937864	no
Mean %GM Untethered	-57%	32%	0.014	0.003846	no	0.003937864	no
Mean %GM D and P1	-71%	27%	0.710	0.003846	no	0.003937864	no
Mean %GM P2 & P1	-48%	32%	0.020	0.003846	no	0.003937864	no
Between Tethered and Untethered			5.12E-11	0.003846	yes	0.003937864	yes
Mean %GM Dd	-74%	27%	0.006	0.003846	no	0.003937864	no
Mean %GM D1	0%	31%	0.510	0.003846	no	0.003937864	no
Mean %GM D2	21%	22%	0.201	0.003846	no	0.003937864	no
Mean %GM D3	14%	17%	0.375	0.003846	no	0.003937864	no
Mean %GM D4	15%	28%	0.291	0.003846	no	0.003937864	no
Mean %GM D5	55%	25%	0.389	0.003846	no	0.003937864	no
Mean %GM P1	-68%	30%	0.171	0.003846	no	0.003937864	no
Mean %GM P2	-28%	18%	0.376	0.003846	no	0.003937864	no

Mean Vertebral Growth Rates

	Mean Growth Rates	STD Total Thickness	p value	Bonf α	Bonf sig	Dunn-Sidàk α	sig
Mean Vertebral Growth Rate Tethered	82.86774	8.017794	0.020	0.003846	no	0.003937864	no
Mean Vertebral Growth Rate Untethered	75.6855	9.899667	0.570	0.003846	no	0.003937864	no
Mean Vertebral Growth Rate D and P1	74.00394	11.1001	0.718	0.003846	no	0.003937864	no
Mean Vertebral Growth Rate P2 & P1	75.90554	11.91532	0.386	0.003846	no	0.003937864	no
Between Tethered and Untethered			0.008	0.003846	no	0.003937864	no
Mean Vertebral Growth Rate Dd	75.24542	4.529703	0.485	0.003846	no	0.003937864	no
Mean Vertebral Growth Rate D1	89.9021	9.936305	0.292	0.003846	no	0.003937864	no
Mean Vertebral Growth Rate D2	86.56952	5.678298	0.161	0.003846	no	0.003937864	no
Mean Vertebral Growth Rate D3	82.00834	8.342324	0.040	0.003846	no	0.003937864	no
Mean Vertebral Growth Rate D4	78.09958	5.225833	0.025	0.003846	no	0.003937864	no
Mean Vertebral Growth Rate D5	77.75914	3.109295	0.650	0.003846	no	0.003937864	no
Mean Vertebral Growth Rate P1	72.76246	15.71146	0.332	0.003846	no	0.003937864	no
Mean Vertebral Growth Rate P2	79.04862	6.464162	0.976	0.003846	no	0.003937864	no

Prox vs Distal Mean Growth Rates

	Distal Physis							Proximal Physis						
	Mean Growth Rate	STD Total Thickness	p value	Bonf α	Bonf sig	Dunn-Sidàk α	sig	Mean Growth Rate	STD Total Thickness	p value	Bonf α	Bonf sig	Dunn-Sidàk α	sig
Mean Growth Rate Tethered	84.83329	5.940162	0.089	0.003846	no	0.003937864	no	80.90219	9.465603	0.015	0.003846	no	0.003937864	no
Mean Growth Rate Untethered	78.45637	11.43229	0.960	0.003846	no	0.003937864	no	72.91463	7.764345	0.087	0.003846	no	0.003937864	no
Mean Growth Rate D and P1	78.20746	13.49951	0.821	0.003846	no	0.003937864	no	69.80042	6.813465	0.174	0.003846	no	0.003937864	no
Mean Growth Rate P2 & P1	79.31987	14.11961	0.957	0.003846	no	0.003937864	no	72.49121	9.219713	0.061	0.003846	no	0.003937864	no
Between Tethered and Untethered			0.084	0.003846	no	0.003937864	no			0.044	0.003846	no		no
Mean Growth Rate Dd	76.72938	4.203592	0.485	0.003846	no	0.003937864	no	73.76146	5.197974					
Mean Growth Rate D1	85.19921	5.169942	0.292	0.003846	no	0.003937864	no	94.60499	12.39942					
Mean Growth Rate D2	89.94487	6.459403	0.161	0.003846	no	0.003937864	no	83.19418	2.169086					
Mean Growth Rate D3	88.34194	5.995354	0.040	0.003846	no	0.003937864	no	75.67475	4.206936					
Mean Growth Rate D4	82.24545	3.279564	0.025	0.003846	no	0.003937864	no	73.95371	2.439875					
Mean Growth Rate D5	78.43496	1.59191	0.650	0.003846	no	0.003937864	no	77.08333	4.50166					
Mean Growth Rate P1	79.68554	20.76939	0.332	0.003846	no	0.003937864	no	65.83938	6.478419					
Mean Growth Rate P2	78.9542	8.163289	0.976	0.003846	no	0.003937864	no	79.14304	6.147961					

Prox vs Distal % Growth Modulation

	Distal Physis							Proximal Physis						
	Mean % Growth Modulation	STD Total Thickness	p value	Bonf α	Bonf sig	Dunn-Sidàk α	sig	Mean % Growth Modulation	STD Total Thickness	p value	Bonf α	Bonf sig	Dunn-Sidàk α	sig
Mean %GM Tethered	23%	33%	0.085	0.003846	no	0.003937864	no	19%	27%	0.972	0.003846	no	0.003937864	no
Mean %GM Untethered	-58%	31%	0.122	0.003846	no	0.003937864	no	-56%	35%	0.000	0.003846	yes	0.003937864	yes
Mean %GM D and P1	-69%	30%	0.184	0.003846	no	0.003937864	no	-74%	27%	0.006	0.003846	no	0.003937864	no
Mean %GM P2 & P1	-61%	39%	0.119	0.003846	no	0.003937864	no	-35%	17%	0.005	0.003846	no	0.003937864	no
Between Tethered and Untethered			6.28E-06	0.003846	yes	0.003937864	yes			5.73E-06	0.003846	yes	0.003937864	yes
Mean %GM Dd	-52%	2%	0.006	0.003846	no	0.003937864	no	-97%	15%					
Mean %GM D1	9%	46%	0.510	0.003846	no	0.003937864	no	-10%	5%					
Mean %GM D2	33%	19%	0.201	0.003846	no	0.003937864	no	9%	20%					
Mean %GM D3	7%	24%	0.375	0.003846	no	0.003937864	no	20%	2%					
Mean %GM D4	2%	20%	0.291	0.003846	no	0.003937864	no	29%	32%					
Mean %GM D5	65%	15%	0.389	0.003846	no	0.003937864	no	45%	32%					
Mean %GM P1	-86%	37%	0.171	0.003846	no	0.003937864	no	-50%	3%					
Mean %GM P2	-35%	25%	0.376	0.003846	no	0.003937864	no	-21%	8%					

Low Tension

Nucleus Pulposis Location — alpha 0.05; k 6 Reported p-values (both blocks)

File: EXP 23101 Bioquant Data
Found in 'Low Tensions NP Location' tab

	%=distance Posterior VB to midpoint NP							% distance Posterior **ossified** epiphysis to midpoint NP						
	Mean	STD	P-value	Bonf α	Bonf sig	Dunn-Sidàk α	sig	Mean	STD	P-value	Bonf α	Bonf sig	Dunn-Sidàk α	sig
Mean % Across All Tethered	48.77%	3.91%	0.490	0.008333	no	0.008512445	no	42.26%	3.91%	0.105	0.008333	no	0.008512445	no
Mean % Across all Untethered	44.99%	3.83%	0.056	0.008333	no	0.008512445	no	36.67%	1.97%	0.159	0.008333	no	0.008512445	no
Mean %Across D and P1	45.66%	4.38%	0.023	0.008333	no	0.008512445	no	35.98%	1.96%	0.039	0.008333	no	0.008512445	no
Mean % Across P2	43.31%	2.66%	0.133	0.008333	no	0.008512445	no	36.75%	1.95%	0.483	0.008333	no	0.008512445	no
Between Tethered and Untethered			0.004	0.008333	yes	0.008512445	yes			0.585	0.008333	no	0.008512445	no
Mean % across Dd	48.33%	3.99%	0.186	0.008333	no	0.008512445	no	36.52%	2.45%	0.141	0.008333	no	0.008512445	no
Mean % Across D1	48.14%	3.17%	0.376	0.008333	no	0.008512445	no	43.18%	2.36%	0.232	0.008333	no	0.008512445	no
Mean % Across D2 (NA)														
Mean % Across D3	50.87%	5.49%	0.414	0.008333	no	0.008512445	no	44.58%	4.03%	0.215	0.008333	no	0.008512445	no
Mean % Across D4	47.30%	3.24%	0.063	0.008333	no	0.008512445	no	39.03%	3.76%	0.386	0.008333	no	0.008512445	no
Mean % Across D5 (NA)														
Mean % Across P1	42.99%	3.27%	0.009	0.008333	no	0.008512445	no	35.44%	1.64%	0.044	0.008333	no	0.008512445	no
Mean % Across P2	43.64%	2.58%	0.385	0.008333	no	0.008512445	no	38.05%	1.29%	0.213	0.008333	no	0.008512445	no

Epiphyseal Percent Ossification — File: EXP 23101 Bioquant Data; alpha 0.05; k 8 Reported p-values (both blocks)
Define — Found in 'Low Tension %Epiphyse

% Epiphyseal Ossification	Mean Low	STD	P-value	Bonf α	Bonf sig	Dunn-Sidàk α	sig	Mean High	STD	P-value	Bonf α	Bonf sig	Dunn-Sidàk α	sig
Mean % Across All Tethered	86.705%	4.64%	0.002	0.00625	yes	0.006391151	yes	93.21%	6.19%					
Mean % Across all Untethered	81.831%	5.46%	0.018	0.00625	no	0.006391151	no	78.95%	5.31%					
Mean %Across D and P1	79.096%	4.24%	0.075	0.00625	no	0.006391151	no	76.36%	3.54%					
Mean % Across P2 & P1	84.936%	3.48%	0.002	0.00625	yes	0.006391151	yes	80.52%	5.09%					
Between Tethered and Untethered			7.02E-05	0.00625	yes	0.006391151	yes							
Mean % across Dd and D1	75.621%	1.45%	0.469	0.00625	no	0.006391151	no	75.81%	5.08%					
Mean % Across D1	89.768%	2.42%	0.042	0.00625	no	0.006391151	no	95.41%	3.62%					
Mean % Across D2 (NA)														
Mean % Across D3	87.771%	1.98%	0.008	0.00625	no	0.006391151	no	96.24%	1.87%					
Mean % Across D4	82.577%	6.78%	0.184	0.00625	no	0.006391151	no	87.99%	8.64%					
Mean % Across D5 (NA)														
Mean % Across P1	82.570%	2.56%	0.001	0.00625	yes	0.006391151	yes	76.91%	2.14%					
Mean % Across P2	87.302%	2.65%	0.092	0.00625	no	0.006391151	no	84.14%	4.57%					

Growth Plate Analysis
Low tension: File: EXP 23101 Bioquant Data; Found in 'Low Tension GP Analysi; alpha 0.05; k 12 Reported p-values
High tension: File: EXP 23101 Bioquant Data; Found in 'GP Analysis' tab; alpha 0.05; k 0 Reported p-values

	Low Tension Mean Thickness	STD Total Thickness	p value	Bonf α	Bonf sig	Dunn-Sidàk α	sig	High Tension Mean Thickness	STD Total Thickness	p value	Bonf α	Bonf sig	Dunn-Sidàk α	sig
Mean Total Thickness Tethered	627.170	97.241	0.322	0.003846	no	0.003937864	no	615.645	95.368					
Mean Total Thickness All Untethered	733.279	123.279	0.416	0.003846	no	0.003937864	no	724.203	99.222					
Mean Thickness D and P1	688.361	63.397	0.289	0.003846	no	0.003937864	no	718.142	109.728					
Mean Thickness P2 & P1	760.733	142.384	0.415	0.003846	no	0.003937864	no	749.600	88.600					
Between Tethered and Untethered														
Mean Total Thickness Dd	678.370	57.654	0.481	0.003846	no	0.003937864	no	673.409	118.110					
Mean Total Thickness D1	665.015	137.238	0.420	0.003846	no	0.003937864	no	648.854	99.612					
Mean Total Thickness D2	581.697	118.819	0.397	0.003846	no	0.003937864	no	569.512	129.978					
Mean Total Thickness D3	597.779	135.519	0.385	0.003846	no	0.003937864	no	580.575	62.236					
Mean Total Thickness D4	628.963	71.758	0.317	0.003846	no	0.003937864	no	651.518	85.351					
Mean Total Thickness D5	662.395	36.215	0.375	0.003846	no	0.003937864	no	627.768	129.801					
Mean Total Thickness P1	698.352	80.152	0.169	0.003846	no	0.003937864	no	762.875	100.735					
Mean Total Thickness P2	823.114	180.513	0.146	0.003846	no	0.003937864	no	736.324	94.598					

Repeat with Proliferative Zone Values
File: EXP 23101 Bioquant Data; Found in 'Proliferative Zone Analysis' tab; Area marked in GREEN. See image to the right. (both blocks)

	Low Tension Mean Prolif Zone	STD Total Thickness	p value	Bonf α	Bonf sig	Dunn-Sidàk α	sig	High Tension Mean Prolif Zone	STD Total Thickness	p value	Bonf α	Bonf sig	Dunn-Sidàk α	sig
Mean Proliferative Thickness Tethered	385.403	43.380	0.4806	0.003846	no	0.003937864	no	371.147	35.355					
Mean Proliferative Thickness All Untethered	449.770	63.328	0.0323	0.003846	no	0.003937864	no	414.949	34.683					
Mean Proliferative Thickness D and P1	449.770	63.328	0.1962	0.003846	no	0.003937864	no	422.120	40.544					
Mean Proliferative Thickness P2 & P1	488.985	70.533	0.0103	0.003846	no	0.003937864	no	409.688	31.364					
Between Tethered and Untethered														
Mean Proliferative Thickness Dd	410.555	20.920	0.2056	0.003846	no	0.003937864	no	425.470	45.862					
Mean Total Proliferative Thickness D1	385.403	43.380	0.3511	0.003846	no	0.003937864	no	374.364	16.959					
Mean Total Proliferative Thickness D2	352.236	20.656	0.2061	0.003846	no	0.003937864	no	380.922	32.227					
Mean Total Proliferative Thickness D3	356.977	46.311	0.2633	0.003846	no	0.003937864	no	381.606	58.103					
Mean Total Proliferative Thickness D4	366.049	21.683	0.2392	0.003846	no	0.003937864	no	341.548	48.466					
Mean Total Proliferative Thickness D5	398.074	39.762	0.2404	0.003846	no	0.003937864	no	377.293	2.248					
Mean Total Proliferative Thickness P1	488.985	70.533	0.1421	0.003846	no	0.003937864	no	418.770	44.413					
Mean Total Proliferative Thickness P2	488.205	37.722	0.0225	0.003846	no	0.003937864	no	400.607	15.471					

Repeat with Hypertrophic Zone Values
File: EXP 23101 Bioquant Data; Found in 'Hypertrophic Zone Analysis' tab; Area marked in RED. See image to the right. (both blocks)

	Mean Total Thickness	STD Total Thickness	p value	Bonf α	Bonf sig	Dunn-Sidàk α	sig	Mean Total Thickness	STD Total Thickness	p value	Bonf α	Bonf sig	Dunn-Sidàk α	sig
Mean Hypertrophic Thickness Tethered	252.218	16.676	0.000	0.003846	yes	0.003937864	yes	281.896	27.839					
Mean Hypertrophic Thickness All Untethered	252.799	73.072	0.303	0.003846	no	0.003937864	no	272.606	23.540					
Mean Hypertrophic Thickness D and P1	230.488	40.190	0.141	0.003846	no	0.003937864	no	268.335	26.226					
Mean Hypertrophic Thickness P2 & P1	257.114	87.103	0.279	0.003846	no	0.003937864	no	279.430	26.117					
Between Tethered and Untethered														
Mean Total Hypertrophic Thickness Dd	244.170	47.147	0.341	0.003846	no	0.003937864	no	258.958	9.598					
Mean Total Hypertrophic Thickness D1	255.644	7.927	0.066	0.003846	no	0.003937864	no	289.667	19.420					
Mean Total Hypertrophic Thickness D2	266.606	24.380	0.153	0.003846	no	0.003937864	no	285.564	47.093					
Mean Total Hypertrophic Thickness D3	247.991	25.787	0.018	0.003846	no	0.003937864	no	290.181	34.339					
Mean Total Hypertrophic Thickness D4	240.050	3.769	0.037	0.003846	no	0.003937864	no	279.279	16.952					
Mean Total Hypertrophic Thickness D5	250.798	5.491	0.248	0.003846	no	0.003937864	no	264.790	26.472					
Mean Total Hypertrophic Thickness P1	216.806	35.409	0.096	0.003846	no	0.003937864	no	277.712	36.928					
Mean Total Hypertrophic Thickness P2	297.421	113.312	0.406	0.003846	no	0.003937864	no	281.149	18.241					

% Growth Modulation
File: Stats.GR.Prox vs Distal Physis.Longtether
Found in 'Low Tension %GM' tab

	Mean % Growth Modulation	STD Total Thickness	p value	Bonf α	Bonf sig	Dunn-Sidàk α	sig	Mean % Growth Modulation	STD Total Thickness	p value	Bonf α	Bonf sig	Dunn-Sidàk α	sig
Mean %GM Tethered	25%	24%	0.210	0.003846	no	0.003937864	no	21%	30%					
Mean %GM Untethered	-41%	23%	0.044	0.003846	no	0.003937864	no	-57%	32%					
Mean %GM D and P1	-48%	24%	0.026	0.003846	no	0.003937864	no	-71%	27%					
Mean %GM P2 & P1	-40%	26%	0.248	0.003846	no	0.003937864	no	-48%	32%					
Between Tethered and Untethered														
Mean %GM Dd	-43%	16%	0.006	0.003846	no	0.003937864	no	-74%	27%					
Mean %GM D1	14%	16%	0.118	0.003846	no	0.003937864	no	0%	31%					
Mean %GM D2	10%	19%	0.222	0.003846	no	0.003937864	no	21%	22%					
Mean %GM D3	22%	16%	0.269	0.003846	no	0.003937864	no	14%	17%					
Mean %GM D4	27%	18%	0.133	0.003846	no	0.003937864	no	15%	28%					
Mean %GM D5	55%	24%	0.495	0.003846	no	0.003937864	no	55%	25%					
Mean %GM P1	-53%	31%	0.246	0.003846	no	0.003937864	no	-68%	30%					
Mean %GM P2	-27%	12%	0.485	0.003846	no	0.003937864	no	-28%	18%					

Mean Vertebral Growth Rates
File: Stats.GR.Prox vs Distal Physis.Longtether
Found in 'Low Tension %GM' tab

	Mean Growth Rates	STD Total Thickness	p value	Bonf α	Bonf sig	Dunn-Sidàk α	sig	Mean Growth Rates	STD Total Thickness	p value	Bonf α	Bonf sig	Dunn-Sidàk α	sig
Mean Vertebral Growth Rate Tethered	81.335	13.60109	0.320	0.003846	no	0.003937864	no	82.86774	8.017794					
Mean Vertebral Growth Rate Untethered	78.3545	12.09306	0.182	0.003846	no	0.003937864	no	75.6855	9.899667					
Mean Vertebral Growth Rate D and P1	78.75988	12.12239	0.108	0.003846	no	0.003937864	no	74.00394	11.1001					
Mean Vertebral Growth Rate P2 & P1	75.97191	13.23076	0.493	0.003846	no	0.003937864	no	75.90554	11.91532					
Between Tethered and Untethered														
Mean Vertebral Growth Rate Dd	83.11969	8.440042	0.082	0.003846	no	0.003937864	no	75.24542	4.529703					
Mean Vertebral Growth Rate D1	88.5989	14.77297	0.417	0.003846	no	0.003937864	no	89.9021	9.936305					
Mean Vertebral Growth Rate D2	86.76621	14.17906	0.493	0.003846	no	0.003937864	no	86.56952	5.678298					
Mean Vertebral Growth Rate D3	82.14399	10.34709	0.494	0.003846	no	0.003937864	no	82.00834	8.342324					
Mean Vertebral Growth Rate D4	76.96249	13.35238	0.454	0.003846	no	0.003937864	no	78.09958	5.225833					
Mean Vertebral Growth Rate D5	72.20343	12.0748	0.301	0.003846	no	0.003937864	no	77.75914	3.109295					
Mean Vertebral Growth Rate P1	74.40008	14.3681	0.402	0.003846	no	0.003937864	no	72.76246	15.71146					
Mean Vertebral Growth Rate P2	77.54373	13.14325	0.391	0.003846	no	0.003937864	no	79.04862	6.464162					

Prox vs Distal Mean Growth Rates
File: Stats.GR.Prox vs Distal Physis.Longtether
Found in 'Low Tension %GM' tab

	Distal Physis							Proximal Physis						
	Mean Growth Rate	STD Total Thickness	p value	Bonf α	Bonf sig	Dunn-Sidàk α	sig	Mean Growth Rate	STD Total Thickness	p value	Bonf α	Bonf sig	Dunn-Sidàk α	sig
Mean Growth Rate Tethered	83.23442	15.0085	0.351	0.003846	no	0.003937864	no	79.43558	12.2559	0.337	0.003846	no	0.003937864	no
Mean Growth Rate Untethered	84.98017	7.971383	0.049	0.003846	no	0.003937864	no	71.72883	12.18392	0.385	0.003846	no	0.003937864	no
Mean Growth Rate D and P1	83.64044	8.70488	0.124	0.003846	no	0.003937864	no	73.87933	13.79684	0.183	0.003846	no	0.003937864	no
Mean Growth Rate P2 & P1	85.22031	9.296092	0.135	0.003846	no	0.003937864	no	66.7235	9.66541	0.133	0.003846	no	0.003937864	no
Between Tethered and Untethered														
Mean Growth Rate Dd	84.49989	6.13266	0.147	0.003846	no	0.003937864	no	81.73949	11.60868	0.082	0.003846	no	0.003937864	no
Mean Growth Rate D1	92.10991	17.97903	0.227	0.003846	no	0.003937864	no	85.08789	13.61522	0.140	0.003846	no	0.003937864	no
Mean Growth Rate D2	89.69646	18.84009	0.494	0.003846	no	0.003937864	no	83.83596	11.04111	0.461	0.003846	no	0.003937864	no
Mean Growth Rate D3	82.15111	10.49746	0.279	0.003846	no	0.003937864	no	82.13687	12.54825	0.272	0.003846	no	0.003937864	no
Mean Growth Rate D4	78.51849	15.34511	0.376	0.003846	no	0.003937864	no	75.40648	14.24708	0.443	0.003846	no	0.003937864	no
Mean Growth Rate D5	73.69615	14.16101	0.327	0.003846	no	0.003937864	no	70.71071	12.54124	0.281	0.003846	no	0.003937864	no
Mean Growth Rate P1	82.78099	12.23159	0.353	0.003846	no	0.003937864	no	66.01917	12.48098	0.491	0.003846	no	0.003937864	no
Mean Growth Rate P2	87.65963	6.969974	0.181	0.003846	no	0.003937864	no	67.42783	8.734265	0.049	0.003846	no	0.003937864	no

Prox vs Distal % Growth Modulation
File: Stats.GR.Prox vs Distal Physis.Longtether
Found in 'Low Tension %GM' tab

	Distal Physis							Proximal Physis						
	Mean % Growth Modulation	STD Total Thickness	p value	Bonf α	Bonf sig	Dunn-Sidàk α	sig	Mean % Growth Modulation	STD Total Thickness	p value	Bonf α	Bonf sig	Dunn-Sidàk α	sig
Mean %GM Tethered	24%	23%	0.490	0.003846	no	0.003937864	no	27%	25%	0.119	0.003846	no	0.003937864	no
Mean %GM Untethered	-51%	25%	0.269	0.003846	no	0.003937864	no	-32%	17%	0.013	0.003846	no	0.003937864	no
Mean %GM D and P1	-61%	22%	0.278	0.003846	no	0.003937864	no	-35%	20%	0.003	0.003846	yes	0.003937864	yes
Mean %GM P2 & P1	-55%	30%	0.367	0.003846	no	0.003937864	no	-25%	10%	0.131	0.003846	no	0.003937864	no
Between Tethered and Untethered														
Mean %GM Dd	-43%	11%	0.121	0.003846	no	0.003937864	no	-44%	23%	0.004	0.003846	yes	0.003937864	yes
Mean %GM D1	12%	14%	0.450	0.003846	no	0.003937864	no	15%	21%	0.107	0.003846	no	0.003937864	no
Mean %GM D2	6%	24%	0.196	0.003846	no	0.003937864	no	14%	18%	0.351	0.003846	no	0.003937864	no
Mean %GM D3	30%	15%	0.194	0.003846	no	0.003937864	no	13%	14%	0.235	0.003846	no	0.003937864	no
Mean %GM D4	19%	12%	0.042	0.003846	no	0.003937864	no	34%	23%	0.350	0.003846	no	0.003937864	no
Mean %GM D5	49%	29%	0.275	0.003846	no	0.003937864	no	60%	22%	0.333	0.003846	no	0.003937864	no
Mean %GM P1	-79%	11%	0.414	0.003846	no	0.003937864	no	-26%	15%	0.072	0.003846	no	0.003937864	no
Mean %GM P2	-30%	19%	0.427	0.003846	no	0.003937864	no	-25%	4%	0.217	0.003846	no	0.003937864	no

High Tension

Nucleus Pulposis Location — alpha 0.05; k 6 Reported p-values (both blocks)

	%=distance Posterior VB to midpoint NP							% distance Posterior **ossified** epiphysis to midpoint NP						
	Mean	STD	P-value	Bonf α	Bonf sig	Dunn-Sidàk α	sig	Mean	STD	P-value	Bonf α	Bonf sig	Dunn-Sidàk α	sig
Mean % Across All Tethered	42.20%	5.72%						45.31%	5.6%					
Mean % Across all Untethered	34.11%	4.34%						43.12%	3.6%					
Mean %Across D and P1	31.79%	2.49%						41.63%	2.5%					
Mean % Across P2	34.88%	4.75%						43.24%	4.2%					
Between Tethered and Untethered														
Mean % across Dd	32.55%	3.66%						42.87%	2.6%					
Mean % Across D1	41.62%	6.79%						43.56%	6.3%					
Mean % Across D2 (NA)														
Mean % Across D3	43.19%	8.60%						44.78%	8.1%					
Mean % Across D4	41.79%	2.95%						47.61%	2.4%					
Mean % Across D5 (NA)														
Mean % Across P1	31.03%	0.66%						40.38%	2.0%					
Mean % Across P2	38.74%	3.37%						46.10%	4.1%					

Prox vs Distal Mean Growth Rates

	Distal Physis							Proximal Physis						
	Mean Growth Rate	STD Total Thickness	p value	Bonf α	Bonf sig	Dunn-Sidàk α	sig	Mean Growth Rate	STD Total Thickness	p value	Bonf α	Bonf sig	Dunn-Sidàk α	sig
Mean Growth Rate Tethered	84.83329	5.940162						80.90219	9.465603					
Mean Growth Rate Untethered	78.45637	11.43229						72.91463	7.764345					
Mean Growth Rate D and P1	78.20746	13.49951						69.80042	6.813465					
Mean Growth Rate P2 & P1	79.31987	14.11961						72.49121	9.219713					
Between Tethered and Untethered														
Mean Growth Rate Dd	76.72938	4.203592						73.76146	5.197974					
Mean Growth Rate D1	85.19921	5.169942						94.60499	12.39942					
Mean Growth Rate D2	89.94487	6.459403						83.19418	2.169086					
Mean Growth Rate D3	88.34194	5.995354						75.67475	4.206936					
Mean Growth Rate D4	82.24545	3.279564						73.95371	2.439875					
Mean Growth Rate D5	78.43496	1.59191						77.08333	4.50166					
Mean Growth Rate P1	79.68554	20.76939						65.83938	6.478419					
Mean Growth Rate P2	78.9542	8.163289						79.14304	6.147961					

Prox vs Distal % Growth Modulation

	Distal Physis							Proximal Physis						
	Mean % Growth Modulation	STD Total Thickness	p value	Bonf α	Bonf sig	Dunn-Sidàk α	sig	Mean % Growth Modulation	STD Total Thickness	p value	Bonf α	Bonf sig	Dunn-Sidàk α	sig
Mean %GM Tethered	23%	33%						19%	27%					
Mean %GM Untethered	-58%	31%						-56%	35%					
Mean %GM D and P1	-69%	30%						-74%	27%					
Mean %GM P2 & P1	-61%	39%						-35%	17%					
Between Tethered and Untethered														
Mean %GM Dd	-52%	2%						-97%	15%					
Mean %GM D1	9%	46%						-10%	5%					
Mean %GM D2	33%	19%						9%	20%					
Mean %GM D3	7%	24%						20%	2%					
Mean %GM D4	2%	20%						29%	32%					
Mean %GM D5	65%	15%						45%	32%					
Mean %GM P1	-86%	37%						-50%	3%					
Mean %GM P2	-35%	25%						-21%	8%					

Nucleus Pulposis Location

alpha 0.05 | k 10 | Reported p-values

File: EXP 23101 Bioquant Data / Found in 'NP Location' tab	%=distance Posterior VB to midpoint NP: Mean	STD	P-value	Bonf α	Bonf sig	Dunn-Sidàk α	sig	% distance Posterior **ossified** epiphysis to midpoint NP: Mean	STD	P-value	Bonf α	Bonf sig	Dunn-Sidàk α	sig
Mean % Across All Tethered	42.60%	0.045	0.156	0.005	no	0.0051162	no	47.50%	4.4%	0.342	0.005	no	0.0051162	no
Mean % Across all Untethered	35.80%	0.039	0.157	0.005	no	0.0051162	no	44.00%	4.2%	0.076	0.005	no	0.0051162	no
Mean %Across D and P1	38.80%	0.038	0.227	0.005	no	0.0051162	no	45.50%	4.2%	0.037	0.005	no	0.0051162	no
Mean % Across P2 and P1	35.82%	0.036	0.005	0.005	yes	0.0051162	yes	43.28%	3.4%	0.105	0.005	no	0.0051162	no
Mean % across Dd	34.50%	0.035	0.031	0.005	no	0.0051162	no	45.60%	4.2%	0.974	0.005	no	0.0051162	no
Mean % Across D1	42.40%	0.046	0.073	0.005	no	0.0051162	no	45.80%	51.1%	0.864	0.005	no	0.0051162	no
Mean % Across D2 (NA)	NA	NA						NA	NA					
Mean % Across D3	43.90%	0.061	0.009	0.005	no	0.0051162	no	47.80%	7.0%	0.189	0.005	no	0.0051162	no
Mean % Across D4	40.40%	0.034	0.434	0.005	no	0.0051162	no	47.50%	2.5%	0.272	0.005	no	0.0051162	no
Mean % Across D5 (NA)	NA	NA						NA	NA					
Mean % Across P1	33.20%	0.027	0.004	0.005	yes	0.0051162	yes	41.70%	2.8%	0.022	0.005	no	0.0051162	no
Mean % Across P2	38.40%	0.023	0.840	0.005	no	0.0051162	no	44.90%	3.3%	0.698	0.005	no	0.0051162	no

Epiphyseal Percent Ossification
Define

File: EXP 23101 Bioquant Data
Found in 'Epiphyseal Ossification' tab

alpha 0.05 | k 11 | Reported p-values

% Epiphyseal Ossification	Mean	STD	P-value	Bonf α	Bonf sig	Dunn-Sidàk α	sig
Mean % Across All Tethered	89.959%	0.064	0.079568259	0.004545455	no	0.004652172	no
Mean % Across all Untethered	80.391%	0.054	0.000913522	0.004545455	yes	0.004652172	yes
Mean %Across D and P1	77.727%	0.040	0.078345939	0.004545455	no	0.004652172	no
Mean % Across P2 & P1	82.728%	0.048	0.020249567	0.004545455	no	0.004652172	no
Between Tethered and Untethered			2.70563E-05	0.004545455	yes	0.004652172	yes
Mean % across Dd	75.715%	0.033	0.0003	0.004545455	yes	0.004652172	yes
Mean % Across D1	92.589%	0.041	0.0068	0.004545455	no	0.004652172	no
Mean % Across D2 (NA)	NA	NA					
Mean % Across D3	92.007%	0.049	0.0134	0.004545455	no	0.004652172	no
Mean % Across D4	85.281%	0.075	0.9706	0.004545455	no	0.004652172	no
Mean % Across D5 (NA)	NA	NA					
Mean % Across P1	79.738%	0.038	0.0532	0.004545455	no	0.004652172	no
Mean % Across P2	85.719%	0.038	0.8508	0.004545455	no	0.004652172	no

Growth Plate Thickness

File: EXP 23101 Bioquant Data
Found in 'GP Analysis' tab

alpha 0.05 | k 13 | Reported p-values

	Mean Total Thickness	STD Total Thickness	p value	Bonf α	Bonf sig	Dunn-Sidàk α	sig
Mean Total Thickness Tethered	621.407	36.449	0.495	0.00384615	no	0.00393786	no
Mean Total Thickness All Untethered	728.740	51.940	0.267	0.00384615	no	0.00393786	no
Mean Thickness D and P1	703.251	38.696	0.296	0.00384615	no	0.00393786	no
Mean Thickness P2 & P1	755.166	34.723	0.479	0.00384615	no	0.00393786	no
Between Tethered and Untethered			0.001	0.00384615	yes	0.00393786	yes
Mean Total Thickness Dd	675.889	83.168	0.744	0.00384615	no	0.00393786	no
Mean Total Thickness D1	656.934	107.616	0.914	0.00384615	no	0.00393786	no
Mean Total Thickness D2	575.605	111.577	0.043	0.00384615	no	0.00393786	no
Mean Total Thickness D3	589.177	94.785	0.091	0.00384615	no	0.00393786	no
Mean Total Thickness D4	640.240	71.598	0.622	0.00384615	no	0.00393786	no
Mean Total Thickness D5	645.081	87.314	0.703	0.00384615	no	0.00393786	no
Mean Total Thickness P1	730.614	88.756	0.108	0.00384615	no	0.00393786	no
Mean Total Thickness P2	779.719	137.380	0.005	0.00384615	no	0.00393786	no

	Mean Ant Unossified total Thickness	STD	p-value	Bonf α	Bonf sig	Dunn-Sidàk α	sig
Mean Total Thickness Tethered	584.711	42.277	0.325	0.00384615	no	0.00393786	no
Mean Total Thickness All Untethered	641.866	34.497	0.277	0.00384615	no	0.00393786	no
Mean Thickness D and P1	622.222	8.052	0.814	0.00384615	no	0.00393786	no
Mean Thickness P2 & P1	648.841	45.696	0.128	0.00384615	no	0.00393786	no
Between Tethered and Untethered			0.001	0.00384615	yes	0.00393786	yes
Mean Total Thickness Dd	627.916	78.110	0.259	0.00384615	no	0.00393786	no
Mean Total Thickness D1	581.473	135.907	0.977	0.00384615	no	0.00393786	no
Mean Total Thickness D2	482.634	78.483	0.008	0.00384615	no	0.00393786	no
Mean Total Thickness D3	549.701	73.224	0.421	0.00384615	no	0.00393786	no
Mean Total Thickness D4	526.822	65.309	0.157	0.00384615	no	0.00393786	no
Mean Total Thickness D5	584.711	97.426	0.910	0.00384615	no	0.00393786	no
Mean Total Thickness P1	616.528	77.430	0.006	0.00384615	no	0.00393786	no
Mean Total Thickness P2	681.153	55.542	0.343	0.00384615	no	0.00393786	no

	Mean Ant 1/4 Total Thickness	STD	p-value	Bonf α	Bonf sig	Dunn-Sidàk α	sig
Mean Total Thickness Tethered	616.845	41.651	0.477	0.00384615	no	0.00393786	no
Mean Total Thickness All Untethered	707.727	45.009	0.436	0.00384615	no	0.00393786	no
Mean Thickness D and P1	696.306	57.175	0.246	0.00384615	no	0.00393786	no
Mean Thickness P2 & P1	733.652	4.360	0.940	0.00384615	no	0.00393786	no
Between Tethered and Untethered			0.008	0.00384615	no	0.00393786	no
Mean Total Thickness Dd	655.878	55.891	0.780	0.00384615	no	0.00393786	no
Mean Total Thickness D1	637.411	123.873	0.753	0.00384615	no	0.00393786	no
Mean Total Thickness D2	557.550	108.718	0.036	0.00384615	no	0.00393786	no
Mean Total Thickness D3	594.491	102.270	0.209	0.00384615	no	0.00393786	no
Mean Total Thickness D4	629.569	88.148	0.628	0.00384615	no	0.00393786	no
Mean Total Thickness D5	665.205	110.358	0.773	0.00384615	no	0.00393786	no
Mean Total Thickness P1	736.735	150.702	0.061	0.00384615	no	0.00393786	no
Mean Total Thickness P2	730.569	125.242	0.083	0.00384615	no	0.00393786	no

	Mean middle 1/2 Total Thickness	STD	p value	Bonf α	Bonf sig	Dunn-Sidàk α	sig
Mean Total Thickness Tethered	665.320	34.584	0.785	0.00384615	no	0.00393786	no
Mean Total Thickness All Untethered	759.863	41.928	0.603	0.00384615	no	0.00393786	no
Mean Thickness D and P1	738.972	29.956	0.596	0.00384615	no	0.00393786	no
Mean Thickness P2 & P1	780.900	65.086	0.614	0.00384615	no	0.00393786	no
Between Tethered and Untethered			0.018000	0.00384615	no	0.00393786	no
Mean Total Thickness Dd	717.790	149.682	0.643	0.00384615	no	0.00393786	no
Mean Total Thickness D1	696.360	146.573	0.922	0.00384615	no	0.00393786	no
Mean Total Thickness D2	619.009	146.559	0.116	0.00384615	no	0.00393786	no
Mean Total Thickness D3	637.804	143.681	0.227	0.00384615	no	0.00393786	no
Mean Total Thickness D4	685.395	111.970	0.761	0.00384615	no	0.00393786	no
Mean Total Thickness D5	688.033	84.872	0.799	0.00384615	no	0.00393786	no
Mean Total Thickness P1	760.154	116.381	0.268	0.00384615	no	0.00393786	no
Mean Total Thickness P2	801.645	156.425	0.055	0.00384615	no	0.00393786	no

	Mean Posterior 1/4 Total Thickness	STD	p value	Bonf α	Bonf sig	Dunn-Sidàk α	sig
Mean Total Thickness Tethered	630.724	42.718	0.465500	0.00384615	no	0.00393786	no
Mean Total Thickness All Untethered	781.182	77.146	0.322	0.00384615	no	0.00393786	no
Mean Thickness D and P1	740.298	43.287	0.505	0.00384615	no	0.00393786	no
Mean Thickness P2 & P1	816.929	65.086	0.373	0.00384615	no	0.00393786	no
Between Tethered and Untethered			0.000600000	0.00384615	yes	0.00393786	yes
Mean Total Thickness Dd	709.690	171.012	0.319	0.00384615	no	0.00393786	no
Mean Total Thickness D1	663.818	112.059	0.636	0.00384615	no	0.00393786	no
Mean Total Thickness D2	598.469	140.857	0.108	0.00384615	no	0.00393786	no
Mean Total Thickness D3	574.731	91.524	0.042	0.00384615	no	0.00393786	no
Mean Total Thickness D4	674.229	87.501	0.767	0.00384615	no	0.00393786	no
Mean Total Thickness D5	642.375	103.859	0.401	0.00384615	no	0.00393786	no
Mean Total Thickness P1	770.906	133.423	0.173	0.00384615	no	0.00393786	no
Mean Total Thickness P2	862.951	201.332	0.002	0.00384615	yes	0.00393786	yes

Repeat with Proliferative Zone Values

File: EXP 23101 Bioquant Data
Found in 'Proliferative Zone Analysis' tab
Area marked in GREEN. See image to the right.

	Mean Total Thickness	STD Total Thickness	p value	Bonf α	Bonf sig	Dunn-Sidàk α	sig
Mean Proliferative Thickness Tethered	369.811	67.504	0.370	0.00384615	no	0.00393786	no
Mean Proliferative Thickness All Untethered	435.184	99.101	0.140	0.00384615	no	0.00393786	no
Mean Proliferative Thickness D and P1	429.588	105.842	0.111	0.00384615	no	0.00393786	no
Mean Proliferative Thickness P2 & P1	450.502	111.078	0.696	0.00384615	no	0.00393786	no
Between Tethered and Untethered			0.000050	0.00384615	yes	0.00393786	yes
Mean Total Proliferative Thickness Dd	404.547	75.146	0.296	0.00384615	no	0.00393786	no
Mean Total Proliferative Thickness D1	379.884	66.796	0.412	0.00384615	no	0.00393786	no
Mean Total Proliferative Thickness D2	366.040	59.780	0.138	0.00384615	no	0.00393786	no
Mean Total Proliferative Thickness D3	363.639	83.931	0.178	0.00384615	no	0.00393786	no
Mean Total Proliferative Thickness D4	352.750	63.248	0.032	0.00384615	no	0.00393786	no
Mean Total Proliferative Thickness D5	386.742	63.765	0.661	0.00384615	no	0.00393786	no
Mean Total Proliferative Thickness P1	454.628	136.539	0.004	0.00384615	yes	0.00393786	yes
Mean Total Proliferative Thickness P2	446.376	85.618	0.017	0.00384615	no	0.00393786	no

	Mean Ant Unossified total Thickness	STD	p-value	Bonf α	Bonf sig	Dunn-Sidàk α	sig
Mean Proliferative Thickness Tethered	347.284	70.933	0.375	0.00384615	no	0.00393786	no
Mean Proliferative Thickness All Untethered	409.890	108.455	0.627	0.00384615	no	0.00393786	no
Mean Proliferative Thickness D and P1	398.854	126.025	0.572	0.00384615	no	0.00393786	no
Mean Proliferative Thickness P2 & P1	422.130	121.857	0.788	0.00384615	no	0.00393786	no
Between Tethered and Untethered			0.003245	0.00384615	yes	0.00393786	yes
Mean Total Proliferative Thickness Dd	379.289	59.723	0.792	0.00384615	no	0.00393786	no
Mean Total Proliferative Thickness D1	356.715	51.034	0.495	0.00384615	no	0.00393786	no
Mean Total Proliferative Thickness D2	370.115	49.186	0.959	0.00384615	no	0.00393786	no
Mean Total Proliferative Thickness D3	307.646	60.739	0.097	0.00384615	no	0.00393786	no
Mean Total Proliferative Thickness D4	339.280	95.335	0.352	0.00384615	no	0.00393786	no
Mean Total Proliferative Thickness D5	360.581	86.250	0.473	0.00384615	no	0.00393786	no
Mean Total Proliferative Thickness P1	429.753	67.863	0.160	0.00384615	no	0.00393786	no
Mean Total Proliferative Thickness P2	414.507	163.136	0.018	0.00384615	no	0.00393786	no

	Mean Ant 1/4 Total Thickness	STD	p-value	Bonf α	Bonf sig	Dunn-Sidàk α	sig
Mean Proliferative Thickness Tethered	373.554	52.842	0.599	0.00384615	no	0.00393786	no
Mean Proliferative Thickness All Untethered	362.639	42.657	0.757	0.00384615	no	0.00393786	no
Mean Proliferative Thickness D and P1	360.281	45.764	0.537	0.00384615	no	0.00393786	no
Mean Proliferative Thickness P2 & P1	362.320	44.948	0.522	0.00384615	no	0.00393786	no
Between Tethered and Untethered			0.352	0.00384615	no	0.00393786	no
Mean Total Proliferative Thickness Dd	367.372	46.478	0.911	0.00384615	no	0.00393786	no
Mean Total Proliferative Thickness D1	374.243	54.297	0.782	0.00384615	no	0.00393786	no
Mean Total Proliferative Thickness D2	387.994	53.502	0.156	0.00384615	no	0.00393786	no
Mean Total Proliferative Thickness D3	377.040	65.433	0.766	0.00384615	no	0.00393786	no
Mean Total Proliferative Thickness D4	351.487	51.886	0.183	0.00384615	no	0.00393786	no
Mean Total Proliferative Thickness D5	377.280	36.214	0.745	0.00384615	no	0.00393786	no
Mean Total Proliferative Thickness P1	353.899	44.607	0.295	0.00384615	no	0.00393786	no
Mean Total Proliferative Thickness P2	367.617	46.615	0.921	0.00384615	no	0.00393786	no

	Mean middle 1/2 Total Thickness	STD	p value	Bonf α	Bonf sig	Dunn-Sidàk α	sig
Mean Proliferative Thickness Tethered	399.621	66.441	0.060	0.00384615	no	0.00393786	no
Mean Proliferative Thickness All Untethered	461.229	66.326	0.398	0.00384615	no	0.00393786	no
Mean Proliferative Thickness D and P1	463.565	70.573	0.210	0.00384615	no	0.00393786	no
Mean Proliferative Thickness P2 & P1	468.993	66.491	0.420	0.00384615	no	0.00393786	no
Between Tethered and Untethered			0.000064	0.00384615	yes	0.00393786	yes
Mean Total Proliferative Thickness Dd	438.909	64.744	0.281	0.00384615	no	0.00393786	no
Mean Total Proliferative Thickness D1	387.131	59.818	0.140	0.00384615	no	0.00393786	no
Mean Total Proliferative Thickness D2	367.786	49.627	0.020042	0.00384615	no	0.00393786	no
Mean Total Proliferative Thickness D3	432.681	89.924	0.66783	0.00384615	no	0.00393786	no
Mean Total Proliferative Thickness D4	383.915	52.915	0.10673	0.00384615	no	0.00393786	no
Mean Total Proliferative Thickness D5	426.593	56.736	0.85977	0.00384615	no	0.00393786	no
Mean Total Proliferative Thickness P1	456.982	60.858	0.01357	0.00384615	no	0.00393786	no
Mean Total Proliferative Thickness P2	480.003	72.101	0.21082	0.00384615	no	0.00393786	no

	Mean Posterior 1/4 Total Thickness	STD	p value	Bonf α	Bonf sig	Dunn-Sidàk α	sig
Mean Proliferative Thickness Tethered	353.245	74.163	0.038831	0.00384615	no	0.00393786	no
Mean Proliferative Thickness All Untethered	516.367	123.251	0.305	0.00384615	no	0.00393786	no
Mean Proliferative Thickness D and P1	512.025	137.627	0.175	0.00384615	no	0.00393786	no
Mean Proliferative Thickness P2 & P1	536.821	125.715	0.699	0.00384615	no	0.00393786	no
Between Tethered and Untethered			0.0000	0.00384615	yes	0.00393786	yes
Mean Total Proliferative Thickness Dd	460.119	102.908	0.236	0.00384615	no	0.00393786	no
Mean Total Proliferative Thickness D1	401.445	94.040	0.781	0.00384615	no	0.00393786	no
Mean Total Proliferative Thickness D2	338.425	75.828	0.173223	0.00384615	no	0.00393786	no
Mean Total Proliferative Thickness D3	320.736	63.768	0.029	0.00384615	no	0.00393786	no
Mean Total Proliferative Thickness D4	331.256	45.093	0.054	0.00384615	no	0.00393786	no
Mean Total Proliferative Thickness D5	373.128	62.965	0.351	0.00384615	no	0.00393786	no
Mean Total Proliferative Thickness P1	546.628	150.704	0.001	0.00384615	yes	0.00393786	yes
Mean Total Proliferative Thickness P2	525.053	93.999	0.015	0.00384615	no	0.00393786	no

Repeat with Hypertrophic Zone Values

File: EXP 23101 Bioquant Data
Found in 'Hypertrophic Zone Analysis' tab
Area marked in RED. See image to the right.

	Mean Total Thickness	STD Total Thickness	p value	Bonf α	Bonf sig	Dunn-Sidàk α	sig
Mean Hypertrophic Thickness Tethered	267.057	34.681	0.646	0.00384615	no	0.00393786	no
Mean Hypertrophic Thickness All Untethered	262.065	64.140	0.185	0.00384615	no	0.00393786	no
Mean Hypertrophic Thickness D and P1	246.511	47.290	0.936	0.00384615	no	0.00393786	no
Mean Hypertrophic Thickness P2 & P1	268.272	71.553	0.154	0.00384615	no	0.00393786	no
Between Tethered and Untethered			0.627	0.00384615	no	0.00393786	no
Mean Total Hypertrophic Thickness Dd	245.512	36.231	0.354	0.00384615	no	0.00393786	no
Mean Total Hypertrophic Thickness D1	272.655	26.936	0.630	0.00384615	no	0.00393786	no
Mean Total Hypertrophic Thickness D2	276.085	41.041	0.477	0.00384615	no	0.00393786	no
Mean Total Hypertrophic Thickness D3	269.086	36.490	0.807	0.00384615	no	0.00393786	no
Mean Total Hypertrophic Thickness D4	259.664	35.696	0.701	0.00384615	no	0.00393786	no
Mean Total Hypertrophic Thickness D5	257.794	33.983	0.611	0.00384615	no	0.00393786	no
Mean Total Hypertrophic Thickness P1	289.285	81.435	0.223	0.00384615	no	0.00393786	no
Mean Total Hypertrophic Thickness P2	247.259	55.768	0.107	0.00384615	no	0.00393786	no

	Mean Ant Unossified total Thickness	STD	p-value	Bonf α	Bonf sig	Dunn-Sidàk α	sig
Mean Hypertrophic Thickness Tethered	273.799	55.847	0.782	0.00384615	no	0.00393786	no
Mean Hypertrophic Thickness All Untethered	230.836	79.060	0.337	0.00384615	no	0.00393786	no
Mean Hypertrophic Thickness D and P1	215.714	60.836	0.423	0.00384615	no	0.00393786	no
Mean Hypertrophic Thickness P2 & P1	231.562	85.615	0.173	0.00384615	no	0.00393786	no
Between Tethered and Untethered			0.0002	0.00384615	yes	0.00393786	yes
Mean Total Hypertrophic Thickness Dd	229.019	64.874	0.131	0.00384615	no	0.00393786	no
Mean Total Hypertrophic Thickness D1	256.606	69.553	0.836	0.00384615	no	0.00393786	no
Mean Total Hypertrophic Thickness D2	272.211	40.921	0.368	0.00384615	no	0.00393786	no
Mean Total Hypertrophic Thickness D3	283.570	46.767	0.378	0.00384615	no	0.00393786	no
Mean Total Hypertrophic Thickness D4	275.990	59.118	0.461	0.00384615	no	0.00393786	no
Mean Total Hypertrophic Thickness D5	284.935	58.199	0.184	0.00384615	no	0.00393786	no
Mean Total Hypertrophic Thickness P1	258.054	102.374	0.012	0.00384615	no	0.00393786	no
Mean Total Hypertrophic Thickness P2	205.071	58.602	0.856	0.00384615	no	0.00393786	no

	Mean Ant 1/4 Total Thickness	STD	p-value	Bonf α	Bonf sig	Dunn-Sidàk α	sig
Mean Hypertrophic Thickness Tethered	267.004	52.674	0.373	0.00384615	no	0.00393786	no
Mean Hypertrophic Thickness All Untethered	242.100	95.401	0.074	0.00384615	no	0.00393786	no
Mean Hypertrophic Thickness D and P1	214.122	55.360	0.921	0.00384615	no	0.00393786	no
Mean Hypertrophic Thickness P2 & P1	254.698	103.782	0.062	0.00384615	no	0.00393786	no
Between Tethered and Untethered			0.010	0.00384615	no	0.00393786	no
Mean Total Hypertrophic Thickness Dd	215.506	72.875	0.197	0.00384615	no	0.00393786	no
Mean Total Hypertrophic Thickness D1	256.527	54.712	0.921	0.00384615	no	0.00393786	no
Mean Total Hypertrophic Thickness D2	279.975	40.314	0.403	0.00384615	no	0.00393786	no
Mean Total Hypertrophic Thickness D3	285.089	69.999	0.383	0.00384615	no	0.00393786	no
Mean Total Hypertrophic Thickness D4	268.135	52.960	0.459	0.00384615	no	0.00393786	no
Mean Total Hypertrophic Thickness D5	244.172	35.908	0.569	0.00384615	no	0.00393786	no
Mean Total Hypertrophic Thickness P1	301.167	134.304	0.055	0.00384615	no	0.00393786	no
Mean Total Hypertrophic Thickness P2	212.876	37.486	0.174	0.00384615	no	0.00393786	no

	Mean middle 1/2 Total Thickness	STD	p value	Bonf α	Bonf sig	Dunn-Sidàk α	sig
Mean Hypertrophic Thickness Tethered	259.755	40.790	0.424	0.00384615	no	0.00393786	no
Mean Hypertrophic Thickness All Untethered	273.984	79.944	0.742	0.00384615	no	0.00393786	no
Mean Hypertrophic Thickness D and P1	265.569	72.392	0.924	0.00384615	no	0.00393786	no
Mean Hypertrophic Thickness P2 & P1	277.597	91.535	0.570	0.00384615	no	0.00393786	no
Between Tethered and Untethered			0.263	0.00384615	no	0.00393786	no
Mean Total Hypertrophic Thickness Dd	263.598	29.846	0.869	0.00384615	no	0.00393786	no
Mean Total Hypertrophic Thickness D1	269.528	37.146	0.787	0.00384615	no	0.00393786	no
Mean Total Hypertrophic Thickness D2	268.601	47.136	0.828	0.00384615	no	0.00393786	no
Mean Total Hypertrophic Thickness D3	266.201	31.467	0.937	0.00384615	no	0.00393786	no
Mean Total Hypertrophic Thickness D4	250.089	47.859	0.394	0.00384615	no	0.00393786	no
Mean Total Hypertrophic Thickness D5	244.354	38.029	0.235	0.00384615	no	0.00393786	no
Mean Total Hypertrophic Thickness P1	289.285	93.896	0.906	0.00384615	no	0.00393786	no
Mean Total Hypertrophic Thickness P2	266.883	92.090	0.234	0.00384615	no	0.00393786	no

	Mean Posterior 1/4 Total Thickness	STD	p value	Bonf α	Bonf sig	Dunn-Sidàk α	sig
Mean Hypertrophic Thickness Tethered	263.932	63.948	0.086	0.00384615	no	0.00393786	no
Mean Hypertrophic Thickness All Untethered	281.108	79.317	0.422	0.00384615	no	0.00393786	no
Mean Hypertrophic Thickness D and P1	267.422	71.941	0.926	0.00384615	no	0.00393786	no
Mean Hypertrophic Thickness P2 & P1	286.775	91.174	0.320	0.00384615	no	0.00393786	no
Between Tethered and Untethered			0.273	0.00384615	no	0.00393786	no
Mean Total Hypertrophic Thickness Dd	265.524	27.324	0.761	0.00384615	no	0.00393786	no
Mean Total Hypertrophic Thickness D1	307.962	100.456	0.075	0.00384615	no	0.00393786	no
Mean Total Hypertrophic Thickness D2	263.392	41.713	0.903	0.00384615	no	0.00393786	no
Mean Total Hypertrophic Thickness D3	247.601	41.406	0.239	0.00384615	no	0.00393786	no
Mean Total Hypertrophic Thickness D4	241.653	44.844	0.141	0.00384615	no	0.00393786	no
Mean Total Hypertrophic Thickness D5	259.010	56.476	0.537	0.00384615	no	0.00393786	no
Mean Total Hypertrophic Thickness P1	308.482	89.982	0.882	0.00384615	no	0.00393786	no
Mean Total Hypertrophic Thickness P2	268.687	91.978	0.034	0.00384615	no	0.00393786	no

% Growth Modulation

File: Stats.GR.Prox vs Distal Physis.Longtether
Found in '%GM' tab

	Mean % Growth Modulation	STD Total Thickness	p value	Bonf α	Bonf sig	Dunn-Sidàk α	sig
Mean %GM Tethered	23%	27%	1.6E-05	0.00384615	yes	0.00393786	yes
Mean %GM Untethered	-49%	29%	0.004	0.00384615	no	0.00393786	yes
Mean %GM D and P1	-60%	28%	0.906	0.00384615	no	0.00393786	no
Mean %GM P2 & P1	-44%	29%	0.003	0.00384615	yes	0.00393786	yes
Between Tethered and Untethered			1.3E-21	0.00384615	yes	0.00393786	yes
Mean %GM Dd	-59%	19%	0.0002	0.00384615	yes	0.00393786	yes
Mean %GM D1	7%	16%	0.5124	0.00384615	no	0.00393786	no
Mean %GM D2	16%	18%	0.2238	0.00384615	no	0.00393786	no
Mean %GM D3	18%	11%	0.1764	0.00384615	no	0.00393786	no
Mean %GM D4	21%	20%	0.1170	0.00384615	no	0.00393786	no
Mean %GM D5	55%	17%	0.0001	0.00384615	yes	0.00393786	yes
Mean %GM P1	-60%	18%	0.0002	0.00384615	yes	0.00393786	yes
Mean %GM P2	-28%	12%	0.1351	0.00384615	no	0.00393786	no

Mean Vertebral Growth Rates

File: Stats.GR.Prox vs Distal Physis.Longtether
Found in 'Vertebral GR' tab

	Mean Growth Rates	STD Total Thickness	p value	Bonf α	Bonf sig	Dunn-Sidàk α	sig
Mean Vertebral Growth Rate Tethered	164.20274	20.2577568	0.064	0.00384615	no	0.00393786	no
Mean Vertebral Growth Rate Untethered	154.04	16.3359619	0.469	0.00384615	no	0.00393786	no
Mean Vertebral Growth Rate D and P1	152.76382	18.9877288	0.330	0.00384615	no	0.00393786	no
Mean Vertebral Growth Rate P2 & P1	151.87745	17.5591367	0.377	0.00384615	no	0.00393786	no
Between Tethered and Untethered			0.078	0.00384615	no	0.00393786	no
Mean Vertebral Growth Rate Dd	158.3651	13.97689	0.787	0.00384615	no	0.00393786	no
Mean Vertebral Growth Rate D1	178.501	22.36951	0.013	0.00384615	no	0.00393786	no
Mean Vertebral Growth Rate D2	159.47898	29.61494	0.080	0.00384615	no	0.00393786	no
Mean Vertebral Growth Rate D3	164.15233	14.0703	0.616	0.00384615	no	0.00393786	no
Mean Vertebral Growth Rate D4	155.06207	17.16755	0.477	0.00384615	no	0.00393786	no
Mean Vertebral Growth Rate D5	149.96257	18.27748	0.160	0.00384615	no	0.00393786	no
Mean Vertebral Growth Rate P1	147.16254	22.85875	0.073	0.00384615	no	0.00393786	no
Mean Vertebral Growth Rate P2	156.59235	10.12106	0.612	0.00384615	no	0.00393786	no

Prox vs Distal Mean Growth Rates

File: Stats.GR.Prox vs Distal Physis.Longtether
Found in 'PvD Mean GR' tab

	Distal Physis: Mean Growth Rate	STD Total Thickness	p value	Bonf α	Bonf sig	Dunn-Sidàk α	sig	Proximal Physis: Mean Growth Rate	STD Total Thickness	p value	Bonf α	Bonf sig	Dunn-Sidàk α	sig
Mean Growth Rate Tethered	84.033855	11.2445224	0.173	0.00384615	no	0.00393786	no	80.1688854	10.785365	0.045	0.00384615	no	0.00393786	no
Mean Growth Rate Untethered	81.718271	10.1327695	0.902	0.00384615	no	0.00393786	no	72.3217277	9.9297267	0.110	0.00384615	no	0.00393786	no
Mean Growth Rate D and P1	80.923948	11.19501	0.929	0.00384615	no	0.00393786	no	71.8398746	10.590706	0.047	0.00384615	no	0.00393786	no
Mean Growth Rate P2 & P1	82.270091	11.8065792	0.777	0.00384615	no	0.00393786	no	69.6073563	9.4960074	0.192	0.00384615	no	0.00393786	no
Between Tethered and Untethered			0.478	0.00384615	no	0.00393786	no			0.016	0.00384615	no	0.00393786	no
Mean Growth Rate Dd	80.614633	6.34240838	0.543	0.00384615	no	0.00393786	no	77.7504706	9.1546061					
Mean Growth Rate D1	88.654561	12.4224321	0.873	0.00384615	no	0.00393786	no	89.8464377	12.760114					
Mean Growth Rate D2	89.820664	12.5971302	0.311	0.00384615	no	0.00393786	no	83.515069	7.1251663					
Mean Growth Rate D3	85.246523	8.36387024	0.237	0.00384615	no	0.00393786	no	78.9058073	9.0879294					
Mean Growth Rate D4	80.381974	10.1320423	0.331	0.00384615	no	0.00393786	no	74.6800947	9.1763873					
Mean Growth Rate D5	76.065552	9.37892707	0.693	0.00384615	no	0.00393786	no	73.8970186	9.121524					
Mean Growth Rate P1	81.233263	15.3383957	0.061	0.00384615	no	0.00393786	no	65.9292786	8.8942435					
Mean Growth Rate P2	83.306918	8.29597944	0.077	0.00384615	no	0.00393786	no	73.285434	9.3170684					

Prox vs Distal % Growth Modulation

File: Stats.GR.Prox vs Distal Physis.Longtether
Found in 'PvD %GM' tab

	Distal Physis: Mean % Growth Modulation	STD Total Thickness	p value	Bonf α	Bonf sig	Dunn-Sidàk α	sig	Proximal Physis: Mean % Growth Modulation	STD Total Thickness	p value	Bonf α	Bonf sig	Dunn-Sidàk α	sig
Mean %GM Tethered	23%	28%	0.012	0.00384615	no	0.00393786	no	23%	26%	0.002	0.00384615	yes	0.00393786	yes
Mean %GM Untethered	-54%	28%	0.001	0.00384615	yes	0.00393786	yes	-44%	29%	0.006	0.00384615	no	0.00393786	no
Mean %GM D and P1	-65%	25%	0.008	0.00384615	no	0.00393786	no	-54%	30%	0.061	0.00384615	no	0.00393786	no
Mean %GM P2 & P1	-58%	34%	0.003	0.00384615	yes	0.00393786	yes	-30%	14%	0.055	0.00384615	no	0.00393786	no
Between Tethered and Untethered			3.78E-12	0.00384615	yes	0.00393786	yes			1.33E-10	0.00384615	yes	0.00393786	yes
Mean %GM Dd	-47%	8%	0.131	0.00384615	no	0.00393786	no	-71%	34%					
Mean %GM D1	11%	30%	0.585	0.00384615	no	0.00393786	no	2%	19%					
Mean %GM D2	20%	24%	0.498	0.00384615	no	0.00393786	no	11%	17%					
Mean %GM D3	19%	22%	0.864	0.00384615	no	0.00393786	no	17%	10%					
Mean %GM D4	11%	17%	0.123	0.00384615	no	0.00393786	no	31%	25%					
Mean %GM D5	57%	22%	0.753	0.00384615	no	0.00393786	no	53%	26%					
Mean %GM P1	-83%	25%	0.004	0.00384615	no	0.00393786	no	-38%	17%					
Mean %GM P2	-33%	20%	0.249	0.00384615	no	0.00393786	no	-23%	6%					

Mean Regional Growth Rates

File: Stats.GR.Prox vs Distal Physis.Longtether
Found in 'Combo Regional GR' tab

	Mean Growth Rates	STD Total Thickness	p value	Bonf α	Bonf sig	Dunn-Sidàk α	sig	Mean Ant Unossified	STD	p-value	Bonf α	Bonf sig	Dunn-Sidàk α	sig
Mean Vertebral Growth Rate Tethered	82.10137	10.1288784	0.064	0.00384615	no	0.00393786	no	103.696	15.894	0.205	0.00384615	no	0.00393786	no
Mean Vertebral Growth Rate Untethered	77.02	8.16798094	0.469	0.00384615	no	0.00393786	no	63.058	10.299	0.282	0.00384615	no	0.00393786	no
Mean Vertebral Growth Rate D and P1	76.381911	9.49386442	0.330	0.00384615	no	0.00393786	no	60.680	11.627	0.479	0.00384615	no	0.00393786	no
Mean Vertebral Growth Rate P2 & P1	75.938723	8.77956834	0.377	0.00384615	no	0.00393786	no	62.987	8.516	0.043	0.00384615	no	0.00393786	no
Between Tethered and Untethered			0.078	0.00384615	no	0.00393786	no			1.15E-12	0.00384615	yes	0.00393786	yes
Mean Vertebral Growth Rate Dd	79.182552	6.98844482	0.787	0.00384615	no	0.00393786	no	63.199	14.179	0.005	0.00384615	no	0.00393786	no
Mean Vertebral Growth Rate D1	89.250499	11.1847534	0.013	0.00384615	no	0.00393786	no	109.737	21.627	0.020	0.00384615	no	0.00393786	no
Mean Vertebral Growth Rate D2	86.667866	9.53938433	0.080	0.00384615	no	0.00393786	no	99.831	14.070	0.224	0.00384615	no	0.00393786	no
Mean Vertebral Growth Rate D3	82.076165	7.03515201	0.616	0.00384615	no	0.00393786	no	98.189	12.461	0.299	0.00384615	no	0.00393786	no
Mean Vertebral Growth Rate D4	77.531034	8.58377253	0.477	0.00384615	no	0.00393786	no	96.362	15.201	0.400	0.00384615	no	0.00393786	no
Mean Vertebral Growth Rate D5	74.981286	9.13874082	0.160	0.00384615	no	0.00393786	no	114.361	10.679	0.004	0.00384615	no	0.00393786	no
Mean Vertebral Growth Rate P1	73.581271	11.4293764	0.073	0.00384615	no	0.00393786	no	58.161	9.007	0.001	0.00384615	yes	0.00393786	yes
Mean Vertebral Growth Rate P2	78.296176	5.06052953	0.612	0.00384615	no	0.00393786	no	67.814	4.743	0.024	0.00384615	no	0.00393786	no

	Mean Anterior 1/4	STD	p-value	Bonf α	Bonf sig	Dunn-Sidàk α	sig	Mean Central 1/2	STD	p-value	Bonf α	Bonf sig	Dunn-Sidàk α	sig	Mean Posterior 1/4	STD	p-value	Bonf α	Bonf sig	Dunn-Sidàk α	sig
Mean Vertebral Growth Rate Tethered	90.786	10.872	0.761	0.00384615	no	0.00393786	no	81.510	10.599	0.029	0.00384615	no	0.00393786	no	72.712	14.926	5.20E-05	0.00384615	yes	0.00393786	yes
Mean Vertebral Growth Rate Untethered	59.073	9.054	0.103	0.00384615	no	0.00393786	no	77.179	8.419	0.036	0.00384615	no	0.00393786	no	97.492	16.293	0.111	0.00384615	no	0.00393786	no
Mean Vertebral Growth Rate D and P1	56.375	9.743	0.322	0.00384615	no	0.00393786	no	76.146	9.960	0.039	0.00384615	no	0.00393786	no	102.917	15.631	0.572	0.00384615	no	0.00393786	no
Mean Vertebral Growth Rate P2 & P1	58.963	8.606	0.018	0.00384615	no	0.00393786	no	74.830	9.092	0.092	0.00384615	no	0.00393786	no	93.419	17.550	0.194	0.00384615	no	0.00393786	no
Between Tethered and Untethered			1.19E-13	0.00384615	yes	0.00393786	yes			0.1470	0.00384615	no	0.00393786	no			2.42E-06	0.00384615	yes	0.00393786	yes
Mean Vertebral Growth Rate Dd	59.293	10.757	0.004	0.00384615	no	0.00393786	no	81.876	4.407	0.607	0.00384615	no	0.00393786	no	105.638	10.274	0.001	0.00384615	yes	0.00393786	yes
Mean Vertebral Growth Rate D1	93.228	13.219	0.041	0.00384615	no	0.00393786	no	89.033	8.837	0.015	0.00384615	no	0.00393786	no	87.024	10.057	0.506	0.00384615	no	0.00393786	no
Mean Vertebral Growth Rate D2	92.714	9.817	0.050	0.00384615	no	0.00393786	no	86.750	9.600	0.071	0.00384615	no	0.00393786	no	79.635	11.767	0.754	0.00384615	no	0.00393786	no
Mean Vertebral Growth Rate D3	88.964	8.343	0.157	0.00384615	no	0.00393786	no	80.636	8.150	0.846	0.00384615	no	0.00393786	no	74.719	8.082	0.333	0.00384615	no	0.00393786	no
Mean Vertebral Growth Rate D4	86.103	15.658	0.313	0.00384615	no	0.00393786	no	79.389	9.635	0.898	0.00384615	no	0.00393786	no	69.415	8.728	0.091	0.00384615	no	0.00393786	no
Mean Vertebral Growth Rate D5	92.920	7.226	0.046	0.00384615	no	0.00393786	no	71.744	10.024	0.031	0.00384615	no	0.00393786	no	52.765	10.603	2.12E-05	0.00384615	yes	0.00393786	yes
Mean Vertebral Growth Rate P1	53.457	8.525	0.000	0.00384615	yes	0.00393786	yes	70.416	10.955	0.011	0.00384615	no	0.00393786	no	100.195	20.352	0.013	0.00384615	no	0.00393786	no
Mean Vertebral Growth Rate P2	64.469	4.184	0.040	0.00384615	no	0.00393786	no	79.243	3.883	0.868	0.00384615	no	0.00393786	no	86.643	12.378	0.539	0.00384615	no	0.00393786	no

Alpha 0.05 | K 9

Sagittal Cobb Angle	Mean % Growth Modulation	STD Total Thickness	p value	Bonf α	Bonf sig	Dunn-Sidàk α	sig
Mean Tethered	-10.05	6.29	0.00974	0.00555556	no	0.00568304	no
Mean Untethered	-28.75	10.57	0.04983	0.00555556	no	0.00568304	no
Between Tethered and Untethered			0.00005	0.00555556	yes	0.00568304	yes
Mean Dd	-22.92	8.76	0.44932	0.00555556	no	0.00568304	no
Mean Dd & D1			0.00643	0.00555556	no	0.00568304	no
Mean D1	-5.78	4.02	0.00101	0.00555556	yes	0.00568304	yes
Mean D5	-14.33	5.21	0.27115	0.00555556	no	0.00568304	no
Mean D5 & P1			0.00391	0.00555556	yes	0.00568304	yes
Mean P1	-34.59	9.35	0.00014	0.00555556	yes	0.00568304	yes

% Growth Modulation Across	Mean % Growth Modulation	STD Total Thickness	p value	Bonf α	Bonf sig	Dunn-Sidàk α	sig
Mean %GM Tethered	30.6%	29.7%	0.0005	0.00555556	yes	0.00568304	yes
Mean %GM Untethered	-60.8%	18.1%	0.6982	0.00555556	no	0.00568304	no
Between Tethered and Untethered			0.0000	0.00555556	yes	0.00568304	yes
Mean %GM Dd	-58.7%	19.4%	0.0153	0.00555556	no	0.00568304	no
Mean % GM Dd & D1			0.0006	0.00555556	yes	0.00568304	yes
Mean %GM D1	6.5%	15.8%	0.2536	0.00555556	no	0.00568304	no
Mean %GM D5	54.7%	17.3%	0.0000	0.00555556	yes	0.00568304	yes
Mean % GM D5 & P1			0.0001	0.00555556	yes	0.00568304	yes
Mean %GM P1	-63.0%	18.2%	0.0067	0.00555556	no	0.00568304	no

alpha 0.05 | k 7

% Epiphyseal Ossification	Mean	STD	P-value	Bonf α	Bonf sig	Dunn-Sidàk α	sig
Mean % Across All Tethered	89.959%	0.064	0.079568259	0.007142857	no	0.007300832	no
Mean % Across all Untethered	80.391%	0.054	0.078345939	0.007142857	no	0.007300832	no
Between Tethered and Untethered			2.38247E-06	0.007142857	yes	0.007300832	yes
Mean % across Dd	75.715%	0.033	0.000762336	0.007142857	yes	0.007300832	yes
Mean % Across D1	92.589%	0.041	0.009214584	0.007142857	no	0.007300832	no
Mean % Across D3	92.007%	0.049	0.017362451	0.007142857	no	0.007300832	no
Mean % Across D4	85.281%	0.075	0.944106398	0.007142857	no	0.007300832	no
Mean % Across P1	79.738%	0.038	0.074009508	0.007142857	no	0.007300832	no

alpha 0.05 | k 7 (one box above each of the five column groups below)

	Mean Growth Rates	STD Total Thickness	p value	Bonf α	Bonf sig	Dunn-Sidàk α	sig	Mean Ant Unoss	STD Total Thickness	p value	Bonf α	Bonf sig	Dunn-Sidàk α	sig
Mean Vertebral Growth Rate Tethered	82.10137	10.1288784	0.064	0.007142857	no	0.007300832	no	103.695873	15.894212	0.205	0.007142857	no	0.007300832	no
Mean Vertebral Growth Rate D and P1	76.381911	9.49386442	0.330	0.00714286	no	0.00730083	no	60.6798506	11.627009	0.479	0.00714286	no	0.00730083	no
Between Tethered and Untethered			0.032	0.00714286	no	0.00730083	no			0.000	0.00714286	yes	0.00730083	yes
Mean Vertebral Growth Rate Dd	79.18255169	6.988444817	0.987	0.00714286	no	0.00730083	no	63.1987519	14.17922945	0.024	0.00714286	no	0.00730083	no
Mean Vertebral Growth Rate D1	89.25049917	11.18475341	0.008	0.00714286	no	0.00730083	no	109.7368768	21.62671275	0.022	0.00714286	no	0.00730083	no
Mean Vertebral Growth Rate D5	74.98128551	9.138740817	0.287	0.00714286	no	0.00730083	no	114.3608502	10.67943711	0.005	0.00714286	yes	0.00730083	yes
Mean Vertebral Growth Rate P1	73.58127066	11.42937641	0.153	0.00714286	no	0.00730083	no	58.16094938	9.007437462	0.004	0.00714286	yes	0.00730083	yes

	Mean Ant 1/4	STD Total Thickness	p value	Bonf α	Bonf sig	Dunn-Sidàk α	sig	Mean Central 1/2	STD Total Thickness	p value	Bonf α	Bonf sig	Dunn-Sidàk α	sig	Mean Posterior 1/4	STD Total Thickness	p value	Bonf α	Bonf sig	Dunn-Sidàk α	sig
Mean Vertebral Growth Rate Tethered	90.78581219	10.8716033	0.761	0.007142857	no	0.007300832	no	81.5101647	10.5993743	0.029	0.007142857	no	0.007300832	no	72.7117298	14.9261226	0.000	0.007142857	yes	0.007300832	yes
Mean Vertebral Growth Rate D and P1	56.3747977	9.74264839	0.322	0.00714286	no	0.00730083	no	76.1462929	9.95980791	0.039	0.00714286	no	0.00730083	no	102.916795	15.631161	0.572	0.00714286	no	0.00730083	no
Between Tethered and Untethered			0.000	0.00714286	yes	0.00730083	yes			0.074	0.00714286	no	0.00730083	no			0.000	0.00714286	yes	0.00730083	yes
Mean Vertebral Growth Rate Dd	59.29265929	10.75730335	0.036	0.00714286	no	0.00730083	no	81.87618315	4.406820472	0.382	0.00714286	no	0.00730083	no	105.6382574	10.27391608	0.023	0.00714286	no	0.00730083	no
Mean Vertebral Growth Rate D1	93.22770116	13.21894778	0.010	0.00714286	no	0.00730083	no	89.03309486	8.836823689	0.005	0.00714286	yes	0.00730083	yes	87.02412476	10.05736253	0.945	0.00714286	no	0.00730083	no
Mean Vertebral Growth Rate D5	92.92010731	7.226168285	0.011	0.00714286	no	0.00730083	no	71.74374908	10.02414575	0.107	0.00714286	no	0.00730083	no	52.76544345	10.6032314	0.000	0.00714286	yes	0.00730083	yes
Mean Vertebral Growth Rate P1	53.45693611	8.524626695	0.002	0.00714286	yes	0.00730083	yes	70.41640259	10.95532259	0.049	0.00714286	no	0.00730083	no	100.1953317	20.35201251	0.113	0.00714286	no	0.00730083	no
